# SSR Loci Analysis in Transcriptome and Molecular Marker Development in *Polygonatum sibiricum*

**DOI:** 10.1155/2022/4237913

**Published:** 2022-03-08

**Authors:** Qingwen Yang, Yujie Jiang, Yuping Wang, Ruilian Han, Zongsuo Liang, Qiuling He, Qiaojun Jia

**Affiliations:** ^1^College of Life Science and Medicine, Zhejiang Sci-Tech University, Hangzhou, China; ^2^Key Laboratory of Plant Secondary Metabolism Regulation in Zhejiang Province, Hangzhou, China; ^3^Agricultural and Rural Development Center of Chun'an County, Hangzhou, China

## Abstract

To study the SSR loci information and develop molecular markers, a total of 435,858 unigenes in transcriptome of *Polygonatum sibiricum* were used to explore SSR. The distribution frequency of SSR and the basic characteristics of repeat motifs were analyzed using MISA software, and SSR primers were designed by Primer 3.0 software and then validated by PCR. Moreover, the gene function analysis of SSR Unigene was obtained by Blast. The results showed that 112,728 SSR loci were found in the transcriptome of *Polygonatum sibiricum*, which distributed in 435,858 unigenes with a distribution frequency of 25.86%. Mo-nucleotide and Di-nucleotide repeat were the main types, accounted for 83.83% of all SSRs. The repeat motifs of A/T and AC/GT were the predominant repeat types of Mo-nucleotide and Di-nucleotide, respectively. A total of 113,305 pairs of SSR primers with the potential to produce polymorphism were designed for maker development. One hundred and fifty-four of the 500 randomly selected primers not only produced fragments with expected molecular size but also had high polymorphism, which could accurately separate the tested varieties. The gene function of unigenes containing SSR was mostly related to the molecular function of *Polygonatum sibiricum*. The SSR markers in transcriptome of *Polygonatum sibiricum* show rich type, strong specificity, and high potential of polymorphism, which will benefit the candidate gene mining and marker-assisted breeding. The developed markers can also provide technical methods for molecular identification of intraspecific species of *Polygonatum* Mill. and maker-assisted breeding of superior varieties of *Polygonatum* Mill.

## 1. Introduction

*Polygonatum sibiricum* was a perennial herb in Liliaceae, more than 60 species globally, mainly distributed among the north temperate zone and the north subtropical zone [1]. In China, 31 species of *Polygonatum* Mill. were recorded and only three species (*P. cyrtonema* Hua, *P. kingianum* Coll. et Hemsl., and *P. sibiricum* Red.) were introduced in *Chinese Pharmacopoeia* (2020 edition). With the clarification of the effective components of *Polygonatum*, its medicinal value and economic value have been gradually recognized by the market. However, some pseudo *Polygonatum* and shoddy *Polygonatum* rush into the market, and even some accidental poisoning phenomena occur, which seriously affects the clinical application value of *Polygonatum* [2]. There were abundant germplasm resources of *Polygonatum* Mill. in China. At present, most of them were in wild state. However, unreasonable logging and habitat destruction have become more and more serious, resulting in the loss of *Polygonatum* germplasm resources, and some *Polygonatum* germplasm resources are on the verge of extinction [3]. Therefore, it was necessary to systematically collect and protect *Polygonatum* Mill. germplasm resources, so as to provide effective reference for its germplasm resources, improved variety breeding, classification basis, and protection strategies. The traditional procedure for identifying *Polygonatum* Mill. plants depended on the morphological characteristics such as the length-width ratio of *Polygonatum* leaves, the presence or absence of short hairs on the back of leaves, the length of pedicels, and the upper ends of filaments [4–6]. However, classification within the *Polygonatum* Mill. genus through phenotypic characteristics was blurred because of variations and interspecies hybridization [7].

Molecular markers can reveal the genetic relationship between species and subspecies from the level of genetic material such as DNA and have the advantages of being unaffected by the environment, high heritability, and easy detection [8–10]. In recent years, molecular markers including random amplified polymorphic DNA (RAPD), intersimple sequence repeat (ISSR), and DNA barcoding have been used to study the genetic relationship identify germplasm resources and analyze the genetic diversity of *Polygonatum* Mill. [11–15]. Unfortunately, the current molecular markers did not satisfy the demands for identifying *Polygonatum* Mill., which may be due to the limited type and number of molecular markers [12, 16]. For example, a few accessions identified as *P. cyrtonema* Hua through morphological identification methods could not be determined using *ITS2* and *psbA-trn-H* markers derived [12]. In the current study, only 225 SSR molecular markers and 43 EST-SSR molecular markers were published [11, 17]. As a result, these molecular markers were far from satisfying species identification and genetic diversity analysis of *Polygonatum* Mill. [2]. With the rapid development of high-throughput sequence technology and the continuous reduction of sequence cost, abundant transcriptome data have been used to develop molecule markers in medicinal plants, such as *Panax ginseng* C. A. Meyer, *Glycyrrhiza uralensis*, *Pharbitis purpurea* (L.) Voisgt, etc. [18–23]. Simple repeat sequences (SSR), also known as microsatellites, were mainly tandem repeat sequences of 2 to 5 nucleotides as basic repeat units. Microsatellite markers were codominant markers, which can distinguish homozygotes from heterozygotes, detect multiple alleles, and have the advantages of rich polymorphism, simple operation, reliable results, and good repeatability, etc. [24].

In this study, the transcriptome data of *P. sibiricum* were used to analyze the composition, distribution, and characteristics of EST-SSR loci in *P. sibiricum*. Moreover, the potential EST-SSR markers were designed, and polymorphic markers were preliminarily verified their polymorphism levels in different *Polygonatum* Mill. These molecular markers might provide a powerful tool for interspecific identification, genetic diversity analysis, and genetic map construction in *Polygonatum* Mill.

## 2. Materials and Methods

### 2.1. Experimental Materials

There were 10 *Polygonatum* Mill. germplasms' transcriptome sequences assembled and used for SSR discovery, categorizations, and marker development (Table 1). Genomic DNA was extracted using the modified CTAB method [8–10, 25]. The quality of DNA was detected by 1.5% agarose gel electrophoresis, and the concentration and purity of DNA were detected using Nanodrop2000. Each sample was diluted to 50 ng·*μ*L^−1^ and stored at -20°C.

### 2.2. SSR Extraction from Transcriptome Data and Primer Design

The MISA software was used to search the repeat sequence sites in the *P. sibiricum* transcriptome. The search criteria included the number of repetitions for mono-, di-, tri-, tetra-, penta-, and hexa-nucleotides with repetition times of 10, 6, 5, 5, 5, and 5, respectively. Primers for each SSR were designed using Primer3.0 software. The optimal primer length was 17-27 bp, and the expected product size ranged from 100 bp to 300 bp. Five hundred pairs of primers were randomly selected to validate their polymorphisms in 10 germplasm of *Polygonatum* Mill. (Supplementary Table S1).

### 2.3. PCR Analysis and Nondenatured Polyacrylamide Gel Electrophoresis

Three *Polygonatum* Mill. samples (DH-1-AH, D-1-HN, and HJ-2-HN) were used to optimize annealing temperature. Polymerase chain reaction (PCR) of each sample was performed in 10 *μ*L volume containing 5 *μ*L Premix Taq™ (Takara Biomedical Technology, Beijing, China), 0.3 *μ*L forward primer (20 *μ*M), 0.3 *μ*L reverse primer (20 *μ*M), 1 *μ*L DNA (50 ng·*μ*L^−1^), and 3.4 *μ*L ddH_2_O. PCR amplification was performed using the following steps: initial denaturation at 94°C for 5 min, 45 cycles of denaturation at 94°C for 30 s, optimal gradient annealing for 30 s, and extension at 72°C for 1 min, and finally an elongation step at 72°C for 10 min. The PCR product was detected by 2% agarose electrophoresis, and the primers with clear bands at 50-500 bp were selected to characterize polymorphism among 10 germplasm of *Polygonatum* Mill. The amplified products and DL50 DNA marker (Takara Biomedical Technology, Beijing, China) were electrophoresed on 8% nondenaturing polyacrylamide gels [acrylamide-bisacrylamide (39 : 1), 1 × TBE] in the 1 × TBE buffer system at a voltage of 180 V and a time of 1.5 h. Electrophoresis gels were stained with Cell Red Nucleic acid dye solution.

### 2.4. Data Processing

According to the electrophoretogram, clear and repeatable amplified bands were counted. The amplified fragments of markers were designated as 0 in the absence of a band and 1 in the presence of a band. Based on the characterization of a matrix, POPGENE 1.31 software was used to evaluate population genetic parameters, including the number of alleles (Na) and Shannon Information Index (I). The expected heterozygosity (He) and locus Polymorphism Information Content (PIC) were calculated using CERVUS v3.0 software. The Marker index (MI) = NPB × PICav, where PICav = ∑PICi/NPB (PICi: PIC value of no. i marker; NPB: number of polymorphic bands) [26, 27]. The NTSYS-pc version 2.0 software was used to calculate the genetic distance matrix, and an unweighted pair group method analysis (UPGMA) tree was constructed.

## 3. Results and Analysis

### 3.1. SSR Distribution

A total of 112,728 SSR loci were found in 435,858 unigenes of *P. sibiricum* transcriptome by MISA. The frequency of SSR loci was 25.86%, and the number of SSRs was 165,912. There were six kinds of repeat SSR patterns, and the mono-, di-, and tri-nucleotide repeats accounted for 98.28% of the total. The mononucleotide repeats were the most abundant (93,768) with a proportion of 56.52%, followed by di- (45,318, 27.31%), tri- (23,965, 14.44%), tetra- (1,537, 0.93%), hexa- (819, 0.49%), and penta- (505, 0.3%) nucleotide (Table 2).

### 3.2. SSR Repetition Type and Frequency

**Figure 1:**
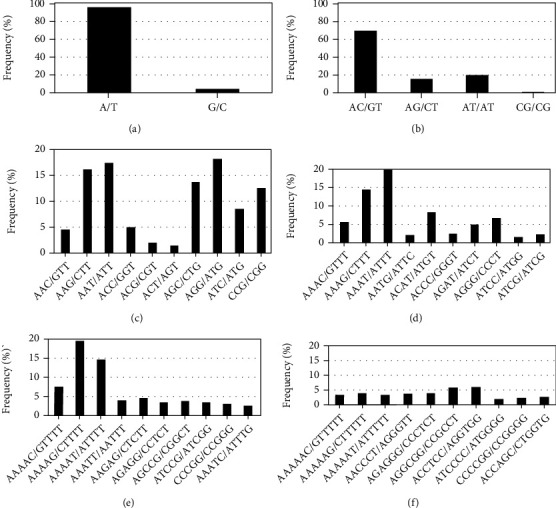
SSR primary types and frequency distribution. Note: (a) repeat type and frequency of single nucleotide; (b) repeat type and frequency of dinucleotide; (c) repeat type and frequency of trinucleotide; (d) repeat type and frequency of tetranucleotide; (e) repeat type and frequency of pentanucleotide; (f) repeat type and frequency of hexanucleotide.

Analysis of repeat loci revealed that among the single nucleotide repeat units, the frequency of A/T was 89,798, accounting for 95.77% of mononucleotide repeats, and G/C was 3,970, accounting for 4.23% (Figure 1(a)). AT/GC balanced repeats were found in the majority of four types of dinucleotide repeat units in *Polygonatum* transcriptome sequences. The most dominant di-nucleotide (AC/GT), accounting for 65.48% of the total SSR repeat, followed by AT/AT (19.12%), AC/GT (14.68%), and CG/CG (0.76%), respectively (Figure 1(b)). Ten types of trinucleotide repeat units were found, among which the frequency of AAG/CTT, AAT/ATT, AGC/CTG, AGG/CTT, and CCG/CGG accounted for 16.20%, 17.46%, 13.82%, 18.17%, and 12.51%, respectively. The frequencies of the other five types were less than 10% (Figure 1(c)). Among the 32 types of tetranucleotides, AAAT/ATTT, AAAG/CTTT, ACAT/ATGT, and AGGG/CCCT were abundantly present exhibiting 36.43%, 14.51%, 8.33%, and 6.70%, respectively (Figure 1(d)). Meanwhile, the most common pena and hexanucleotide repeats were AAAG/CTTTT (19.41%), AAAAT/ATTTT (14.65%), ACCTCC/AGGTGG (6.11%), and AGGCGG/CCGCCT (5.98%), respectively (Figures 1(e) and 1(f)).

### 3.3. Validation of EST-SSR Molecular Markers and Genetic Diversity Analysis in *Polygonatum* Mill.

#### 3.3.1. Polymorphism Analysis of the Newly-Developed EST-SSR Molecular Markers

In order to evaluate the amplification efficiency of the newly developed EST-SSR markers, a total of 500 markers based on the SSR-containing sequence were randomly selected for validation and assessment of the polymorphism in different *Polygonatum* Mill. (DH-1-AN, D-1-HN, and HJ-2-HN). In these selected primer pairs, 241 pairs of primers produced clearly and reproducible amplification products. One hundred and four EST-SSR markers showed polymorphisms and high amplification efficiency among the tested germplasms. Motifs, primer information, and product size of the tested EST-SSRs were listed in Supplementary Table S1.

**Figure 2:**
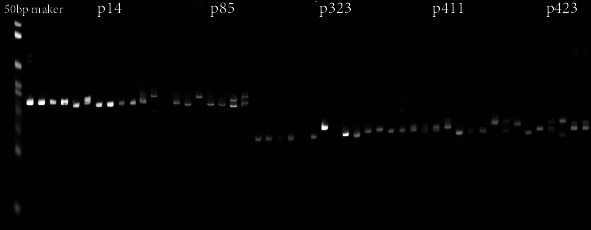
Profile of 10 *Polygonatum* Mill. collections amplified using the primers p14, p85, p323, p411, and p423. The order of 10 *Polygonatum* Mill. germplasm was DH-1-AH, DH-2-GX, DH-3-ZJ, D-1-HN, HJ-4-SX, HJ-1-JX, HJ-2-HN, D-2-GZ, HJ-3-SC, and DH-4-HN.

The polymorphic EST-SSR markers were used to evaluate the genetic diversity of 10 *Polygonatum* Mill. germplasm resources. All primer pairs amplified the fragments and a total of 845 alleles were obtained from 154 EST-SSRs in 10 germplasms. The results of nondenaturing polyacrylamide gels of some primers were shown in Figure 2. The number of alleles (Na) ranged from 3 to 9, with an average of 5.4870. The Shannon Information Index (I) was 0.3406-0.6929, with an average of 0.6177. The Polymorphism Information Content (PIC) value varied from 0.163 to 0.849, with a mean of 0.6005 (PIC > 0.5), which indicated that these loci contained a considerable amount of genetic information and could be used to analyze the genetic diversity of *Polygonatum* Mill. The expected heterozygosity (He) ranged from 0.177 to 0.908, with an average of 0.6740 (Table 3).

#### 3.3.2. Cluster Analysis

**Figure 3:**
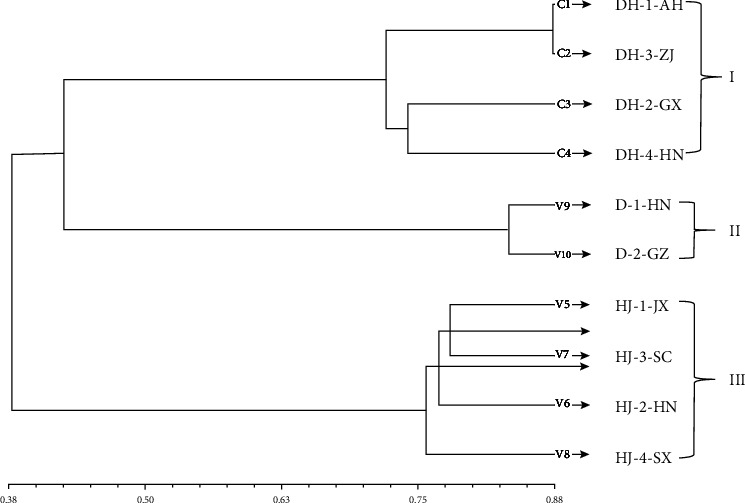
Clustering analysis (UPGMA) of 10 *Polygonatum* germplasm based on SSR markers.

A dendrogram using UPGMA analysis was constructed based on the genetic similarity coefficient of the tested germplasm resources (Figure 3). In the diagram, ten germplasm resources could be divided into three categories when the coefficient was 0.53. All the germplasm resources of *Polygonatum* Mill. were gathered based on species. Cluster I consisted of four *P. cyrtonema* Hua accessions, including DH-1-AH, DH-2-GX, DH-3-ZJ, and DH-4-HN. Group II was comprised of *P. kingianum* Coll. et Hemsl accessions (D1-HN and D2-GZ). All the four *P. sibiricum* Red. accessions (HJ-1-JX, HJ-4-SX, HJ-3-SC, and HJ-2-HN) were concentrated in Group III (Figure 3).

### 3.4. Functional Annotation of SSR-Containing Genes in Transcriptome of *P. sibiricum*

To broaden the functional aspects of SSR marks, all the sequences containing SSR loci were performed gene function annotations in seven public databases, including Nr, Nt, Pfam, KOG/COG, Swiss-prot, KEGG, and GO databases. Among them, 66,396 (58.9%) and 55,861 (50.4%) unigenes were separately annotated to the NR and GO database. Unigenes annotated to NT, Swiss-Prot, and Pfam databases were 48,247 (42.8%), 51,065 (45.3%), and 55,913 (49.3%), respectively. The number of unigenes sequences annotated to the KOG/COG database was the least, only 19,952 (17.7%).

To further understand the function of the SSR-containing genes in *P. sibiricum*, WEGO software was used to annotate the unigenes by GO classification. The results showed that 23,126 (41.4%) of the 55,861 unigenes were categorized in the molecular function, 20,780 (37.2%) of them were related to the biological process, and 14,970 (26.8%) of them were assigned to the cell component category.

## 4. Discussion

### 4.1. The Characteristics of SSR

With the rapid development of sequencing technology, more and more transcriptome data of Chinese herbal medicines such as *S. miltiorrhiza*, *Dendrobium catenatum*, *Polygonatum* Mill., and *G. uralensis* had been released [28], which provided feasibility and practical basis for developing genomic-SSR [29], EST-SSR, SNP [8–10], InDel [30], and KASP molecular markers. In this study, a total of 165,912 SSR loci with a frequency of 25.86% were identified from 435,858 unigenes of *P. sibiricum* transcriptome under the MISA screening conditions. Compared with other plants, the occurrence frequency of SSR loci was higher than that of *P. cyrtonema* Hua (7.89%) and *P. ginseng* C. A. Meyer (7.3%) [19], but lower than that of *G. uralensis* (60.10%) [21] and *Gentiana macrophylla* (30.73%) [23]. It has been reported that bioinformatics software tools, search criteria, and size of the database were used in different studies for identifying microsatellites may result different SSR loci frequencies [31, 32].

With the increase in the number of repeat units, the distribution frequency of genomic SSRs decreased gradually [33], which was consistent with our results (Table 2). We also found that single-nucleotide and dinucleotide repeats were the main repeat types with the most significant number of mononucleotides repeat units (56.52%), followed by dinucleotide distribution units (27.31%). The highest proportion of mononucleotide repeat units was also identified in *G. uralensis* (60.73%) [34], *S. splendens Ker-Gawler* (41.6%) [35], *Punica granatum* L. (51.95%) [36], and *Eucommia ulmoides* (54.34%) [37], whereas dinucleotide repeat units were the dominant motifs in *Fagopyrum tataricum* (L.) *Gaertn* (69.72%) [38], *Rhododendron simsii Planch* (94.58%) [39], and *Gastrodia elata* Bl (78.88%) [40]. In this study, there were 228 types of abundant SSR repeats in *P. sibiricum*. The dominant motifs were A/T (58.28%), AG/CT (10.48%), AT/AT (10.48%), and AC/GT (5.12%). The prevalent of A/T was also identified in *G. uralensis* [34], *G. elata* Bl [40], *P. granatum* L. [36], *P. cyrtonema* Hua, and *E. ulmoides* [37]. Gur-Arie et al. [41] suggested that this phenomenon may be related to the fact that repeat sequences rich in A/T bases are easier to melt in DNA. In addition, this biased result may also be related to the parameter settings in the SSR locus finding tools. Furthermore, the advantage of the di-nucleotide repeat sequence may be attributed to the overexpression of UTRs as compared to open reading frames, according to the previous studies [42, 43]. AG/CT motifs frequently appear in plant EST datasets. Because the AG/CT motif can represent UCU and CUC codons in an mRNA population, which translate to the amino acids Ala and Leu, which are found in proteins at a higher frequency than other amino acids [11].

### 4.2. SSR Primer Validity and Polymorphism

The length of SSR was an important factor affecting its polymorphism. Based on the length of SSR motifs, they can be categorized as low (<12 bp), medium (12-20 bp), or high (≥20 bp). The total number of SSR loci more than 12 bp in the transcriptome of *P. sibiricum* was 105,186, of which 37,290 were more than 20 bp and 67,896 were 12-20 bp. These results indicated that the SSR loci of *P. sibiricum* transcriptome were moderately polymorphic. However, the frequency is thought to be influenced by species differences, as well as the SSR search parameters, database size, and database-mining techniques used in different research.

The polymorphism degree of molecular markers can be measured by the number of alleles (Na), heterozygosity (He), and the Polymorphism Information Content (PIC). A total of 845 alleles were produced by 154 pairs of primers, with an average of 5.4870 alleles per locus. The abundant alleles of the developed markers indicated that EST-SSRs were suitable to detect genetic diversity of the *Polygonatum* Mill. Polymorphism Information Content (PIC) can measure allele frequencies present at single loci or summed multiple loci and act as the discriminatory power of the molecular markers. The degree of PIC values was generally categorized as low (PIC < 0.25), medium (0.5 > PIC > 0.25), or high (PIC > 0.5) [44]. In this study, the average PIC value of the developed markers was 0.6005 (>0.5), which was higher than those of SSR in *Polygonatum* Mill. reported by Zhu et al. [2] and Wang et al. [45]. Therefore, the developed markers exhibited high polymorphism in the tested germplasm. Thus, it was indicated that the newly screened EST-SSR markers were a useful and informative tool for genetic research and evolutionary adaptability across a vast variety of Polygonatum Mill. at the species level. Shannon Information Index (I) and expected heterozygosity (He) also demonstrated that these markers could be used to distinguish these Polygonatum Mill. germplasm well.

### 4.3. Genetic Diversity Analysis

It has been reported that identification within the species of *Polygonatum* Mill. was complicated based on the morphology, possibly due to the interspecific hybridization in *Polygonatum* Mill. [2, 12]. Recently, molecular markers have played an increasingly important role in the identification of *Polygonatum* Mill. species. Most species of the *Polygonatum* Mill. can be identified by molecular markers, while only several accessions identified as *P. cyrtonema* Hua through morphological identification methods could not be determined using markers derived from the chloroplast genome [12], resulting the limitation of mtDNA. Phylogenetic relationship among *Polygonatum* Mill. as revealed by SSR markers was highly consistent with the classification of species. These findings indicated that the newly developed EST-SSR molecular markers could separate the germplasm from different species and accurately reflect the genetic relationship of different germplasm. Although the polymorphism of EST-SSR was lower than that of genomic SSR markers, the sequence derived from the coding region was more conservative and had better universality [8–10]. In addition, the polymorphism of EST-SSR may be directly related to gene function and the identification of germplasm resources by such markers were not affected by environmental factors and material sources. Therefore, EST-SSR molecular markers can provide an effective tool for identifying medicinal plants [11]. These findings not only indicated that *Polygonatum* Mill has active metabolic processes but also that it synthesizes a variety of compounds in this species.

There were many varieties of *Polygonatum* Mill. resources in all parts of the country, which were easy to be mixed, and the prescribed varieties of *Polygonatum* only account for 7.5% of the total varieties of *Polygonatum* Mill. [3]. In this study, these three varieties were selected as samples for the development of molecular markers of *Polygonatum* Mill. Interestingly, the molecular markers we developed can accurately separate these three varieties. This result was undoubtedly a great advance to our research. As far as our current research results are concerned, we can identify these three kinds of *Polygonatum* in circulation in the market efficiently and quickly with the molecular markers we studied. At present, the number of SSR molecular markers reported in the literature can actually have high efficiency and polymorphism at the same time [2], but our research screened out 154 SSR molecular markers with both efficacy, which exceeded the total number of SSR molecular markers reported in the literature at present, and injected a powerful source for the development of *Polygonatum* Mill. molecular markers in the future.

In addition, the numbers of unigenes with SSR in *P. sibiricum* transcriptome were mostly annotated in the molecular function category. Therefore, we speculated that unigenes with SSR site in *P. sibiricum* might be related to molecular function, which pointed out the direction for targeted research on specific gene functions based on SSR in a later stage.

## 5. Conclusion

This study demonstrated comprehensive mining and characterization of specific co-dominant EST-SSR markers using *P. sibiricum* transcriptome. All the tested *Polygonatum* Mill. resources gathered based on species after UPGMA analysis derived from the data matrices of the developed polymorphic EST-SSR markers. Therefore, the developed EST-SSRs offer great potential for the identification of *Polygonatum* Mill. and also facilitating marker-assisted selection in *Polygonatum* Mill. These results would be a valuable resource for future *Polygonatum* Mill. genetic and genomic studies, as well as a potent molecular tool for evolutionary adaption and genetic relationship study in other species.

## Figures and Tables

**Table 1:** Germplasm resources of *Polygonatum* Mill. used in this study.

Population	Sample ID	Geographical origin	Germplasm name
DH	DH-1-AH	Chizhou, Anhui	*Polygonatum cyrtonema* Hua
	DH-2-GX	Hezhou, Guangxi	*Polygonatum cyrtonema* Hua
	DH-3-ZJ	Xianju, Zhejiang	*Polygonatum cyrtonema* Hua
	DH-4-HN	Loudi, Hunan	*Polygonatum cyrtonema* Hua
D	D-1-HN	Lushan, Henan	*Polygonatum kingianum* Coll. et Hemsl
	D-2-GZ	Dejiang, Guizhou	*Polygonatum kingianum* Coll. et Hemsl
HJ	HJ-1-JX	Xinfeng, Jiangxi	*Polygonatum sibiricum* Red.
	HJ-2-HN	Lingbao, Henan	*Polygonatum sibiricum* Red.
	HJ-3-SC	Yaan, Sichuan	*Polygonatum sibiricum* Red.
	HJ-4-SX	Ningqiang, Shanxi	*Polygonatum sibiricum* Red.

**Table 2:** Number and distribution of SSR loci.

Nucleotide types	Repeat times									Total percentage (%)	
	5	6	7	8	9	10	11	12	>12		
Mononucleotide	—	—	—	—	—	37068	16783	10409	29509	93768	56.52
Dinucleotide	—	12538	7561	5599	3698	2385	1734	1267	10536	45318	27.31
Trinucleotide	13077	5616	2470	1223	721	513	71	79	165	23965	14.44
Tetranucleotide	1086	231	128	52	8	3	26	1	2	1537	0.93
Pentanucleotide	394	83	6	5	5	1	—	1	11	505	0.30
Hexanucleotide	670	55	40	9	16	10	1	11	7	819	0.49
Total	15227	18523	10205	6888	4448	39980	18614	11768	40260	165912	100

**Table 3:** Polymorphism analysis of 154 EST-SSRs in 10 *Polygonatum* Mill. germplasm resources.

Loci	Na	I	PIC	He	Loci	Na	I	PIC	He	Loci	Na	I	PIC	He
p2	6	0.5662	0.669	0.749	p164	4	0.6047	0.501	0.616	p319	5	0.5385	0.604	0.7
p6	4	0.6897	0.42	0.489	p171	5	0.6247	0.65	0.739	p323	5	0.6208	0.63	0.714
p7	6	0.5662	0.709	0.784	p172	5	0.6619	0.588	0.662	p324	4	0.6619	0.476	0.574
p10	3	0.4571	0.371	0.464	p173	5	0.6619	0.588	0.662	p326	6	0.6619	0.656	0.732
p17	4	0.5004	0.326	0.363	p175	5	0.6208	0.618	0.7	p334	6	0.6047	0.728	0.805
p21	5	0.6247	0.643	0.732	p178	7	0.5385	0.723	0.791	p336	6	0.5662	0.709	0.784
p22	8	0.4649	0.791	0.853	p180	5	0.5662	0.618	0.708	p341	4	0.673	0.483	0.575
p24	7	0.6208	0.703	0.766	p182	4	0.6926	0.354	0.399	p344	5	0.5385	0.604	0.7
p28	7	0.5385	0.732	0.801	p187	3	0.6845	0.24	0.275	p345	5	0.6619	0.653	0.742
p30	7	0.5004	0.769	0.835	p190	4	0.6929	0.45	0.529	p350	5	0.6247	0.586	0.68
p31	5	0.6897	0.46	0.511	p191	5	0.6816	0.544	0.616	p351	3	0.6926	0.215	0.242
p35	7	0.5385	0.732	0.801	p195	5	0.673	0.428	0.472	p352	7	0.3612	0.797	0.875
p36	6	0.6594	0.656	0.732	p200	5	0.6619	0.562	0.645	p353	6	0.6816	0.65	0.721
p40	6	0.4649	0.709	0.784	p204	4	0.5385	0.52	0.626	p355	6	0.6926	0.683	0.763
p42	5	0.5385	0.65	0.739	p209	4	0.673	0.394	0.455	p357	6	0.5444	0.687	0.765
p45	5	0.6888	0.544	0.616	p210	5	0.6926	0.512	0.576	p360	5	0.5956	0.643	0.732
p46	4	0.5444	0.525	0.642	p214	3	0.6047	0.272	0.318	p361	6	0.6926	0.669	0.749
p57	6	0.5385	0.728	0.805	p215	6	0.6247	0.65	0.721	p364	7	0.5385	0.769	0.835
p61	4	0.6208	0.501	0.616	p220	5	0.6888	0.562	0.645	p365	3	0.4571	0.163	0.177
p63	7	0.673	0.753	0.818	p221	6	0.6619	0.579	0.637	p368	5	0.6845	0.544	0.616
p65	9	0.6247	0.849	0.905	p225	5	0.6247	0.63	0.714	p370	6	0.6452	0.579	0.637
p66	8	0.673	0.81	0.87	p228	6	0.642	0.656	0.732	p371	6	0.6247	0.709	0.784
p71	7	0.5004	0.769	0.835	p231	4	0.6047	0.394	0.455	p374	6	0.5385	0.709	0.784
p72	8	0.673	0.775	0.835	p236	3	0.673	0.272	0.318	p389	5	0.6047	0.618	0.7
p79	8	0.3612	0.81	0.87	p239	5	0.6208	0.643	0.732	p393	8	0.5662	0.835	0.908
p82	9	0.673	0.849	0.905	p241	4	0.5662	0.494	0.61	p394	6	0.673	0.687	0.765
p85	4	0.6926	0.42	0.489	p242	6	0.6888	0.65	0.721	p404	6	0.6047	0.669	0.749
p90	4	0.642	0.394	0.455	p246	4	0.673	0.484	0.593	p405	6	0.6208	0.709	0.784
p108	3	0.5716	0.314	0.378	p247	5	0.6929	0.587	0.67	p411	7	0.5444	0.753	0.818
p109	4	0.6926	0.452	0.541	p248	3	0.5385	0.272	0.318	p416	7	0.6929	0.788	0.853
p111	4	0.5956	0.394	0.455	p256	3	0.6845	0.24	0.275	p420	8	0.6926	0.81	0.87
p113	4	0.6929	0.476	0.574	p260	5	0.6926	0.512	0.576	p421	6	0.5956	0.669	0.749
p116	4	0.673	0.484	0.593	p262	7	0.6047	0.703	0.766	p425	6	0.673	0.746	0.818
p117	5	0.6929	0.562	0.645	p264	5	0.5716	0.653	0.742	p427	8	0.6888	0.81	0.87
p118	7	0.3406	0.753	0.818	p266	5	0.4491	0.672	0.765	p434	6	0.5444	0.752	0.826
p120	5	0.6619	0.562	0.645	p268	5	0.5004	0.653	0.742	p440	8	0.6047	0.727	0.784
p122	6	0.4571	0.737	0.817	p269	4	0.6619	0.484	0.593	p442	5	0.6247	0.59	0.679
p123	4	0.6929	0.484	0.593	p272	5	0.6619	0.562	0.645	p444	6	0.673	0.615	0.68
p125	4	0.6926	0.525	0.642	p274	6	0.6888	0.615	0.68	p448	7	0.6926	0.642	0.697
p127	5	0.5444	0.586	0.68	p278	7	0.4649	0.769	0.835	p452	9	0.6926	0.849	0.905
p129	5	0.6247	0.63	0.714	p282	5	0.5385	0.586	0.68	p463	5	0.673	0.643	0.732
p130	3	0.642	0.194	0.216	p286	3	0.6845	0.24	0.275	p464	5	0.673	0.643	0.732
p132	3	0.673	0.314	0.378	p287	7	0.5385	0.77	0.843	p469	9	0.6247	0.831	0.887
p135	5	0.6816	0.58	0.66	p288	6	0.6619	0.615	0.68	p471	8	0.673	0.81	0.87
p136	4	0.6619	0.484	0.593	p290	5	0.5385	0.643	0.732	p476	7	0.6845	0.753	0.818
p140	4	0.6897	0.452	0.541	p295	7	0.4571	0.778	0.847	p477	9	0.673	0.849	0.905
p149	4	0.6816	0.452	0.541	p298	9	0.6594	0.797	0.853	p483	9	0.673	0.831	0.887
p153	5	0.6208	0.59	0.679	p300	6	0.6594	0.62	0.686	p489	8	0.5004	0.775	0.835
p157	3	0.6816	0.215	0.242	p301	3	0.6208	0.272	0.318	p492	7	0.673	0.807	0.87
p158	5	0.673	0.643	0.732	p309	7	0.6816	0.788	0.853	p499	8	0.6888	0.81	0.87
p161	3	0.6208	0.272	0.318	p315	5	0.4649	0.692	0.803	Sum	845	95.121	92.474	103.79
p163	5	0.6619	0.674	0.763	p316	5	0.6897	0.512	0.576	Mean	5.4870	0.6177	0.6005	0.6740

Na: observed number of alleles; I: Shannon Information Index; PIC: Polymorphism Information Content; He: expected heterozygosity.

## Data Availability

The data that support this study are available in the article and accompanying supplementary material.

## Supplementary Materials

Supplementary Materials Table S1: information of 500 randomly selected primer pairs.

## References

[1] Tian Q. J., Zhao Z. (2007). Species identification and resource distribution of *Polygonatum*. *Research and Practice of Modern Chinese Medicine*.

[2] Zhu Q., Deng X., Zhang S. B. (2018). Genetic diversity of 6 species in *Polygonatum* by SSR marker. *China Journal of Chinese Materia Medica*.

[3] Cui K. S., Xiao T., Li H. P. (2021). Research progress of China’s Polygonatum germplasm resources. *Jiangsu Agricultural Science*.

[4] Hu Y. J., Zhu J. J. (2011). Study on identification of *Polygonatum cyrtonema* and *Polygonatum filipe* in Zhejiang. *Zhejiang Journal of Traditional Chinese Medicine*.

[5] Tang Y. M. (1978). Polygonatum. *Flora Reipublicae Popularis Sinicae*.

[6] Xu H. L., Wang Y. J., Chen M. (2017). Germplasm identification and genetic diversity analysis of *Polygonatum* and *Polygonatum angustifolia* in Fujian Province based on ISSR markers. *Fujian Journal of Agricultural Sciences*.

[7] Yang Q., Gao C. H., Cheng H. L., Chen Q. C., Xu X. J. (2017). Identification of *Polygonatum* from Wuyishan and its surrounding areas by ISSR molecular markers and their HPLC fingerprints. *Subtropical Plant Science*.

[8] Yang B., Cheng M. J., Du Q. X., Zhu J. L., Du H. Y., Yang S. B. (2019). SNP sites developed by whole genome resequencing analysis in *Eucommia ulmoides* 'Hongye'. *Bulletin of Botanical Research*.

[9] Yang L., Wu W. R., Fu F. (2019). Exploration and application of a new method for rapid extraction of DNA from Chinese medicinal materials. *Chinese Traditional and Herbal Drugs*.

[10] Yang M. T., Huang Z., Gan J. P., Xu J. C., Pang J. L. (2019). Advances in SSR molecular markers. *Journal of Hangzhou Normal University(Natural Science Edition)*.

[11] Chen S., Wu G. W., Wu J. Z., Huang W. G., Liu Y., Yang X. (2018). Research progress of EST-SSR in plants. *Heilongjiang Agricultural Sciences*.

[12] Jiao J., Huang W., Bai Z., Liu F., Ma C., Liang Z. (2018). DNA barcoding for the efficient and accurate identification of medicinal *polygonati* rhizoma in China. *PLoS One*.

[13] Wu S. A., Lu H. L., Yang J. (2000). Application of RFLP analysis of chloroplast DNA fragments in systematics of *Polygonatum*. *Journal of Systematics and Evolution*.

[14] Xu S. L. (2019). Analysis and study on identification of *Polygonatum multiflorum* and *Polygonatum pedunculatum* in Xin'an area by RAPD markers. *Journal of Puer University*.

[15] Zhang J., Wang Y. Z., Yang W. Z., Yang M. Q., Zhang J. Y. (2019). Research progress in chemical constituents in plants of *Polygonatum* and their pharmacological effects. *China Journal of Chinese Materia Medica*.

[16] Yang P., Zhou H., Xin T. Y., Ma S. J., Duan B. Z., Yao H. (2015). Identifcation study of DNA barcode sequences in the medicinal plants of *Polygonatum*. *World Chinese Medicine*.

[17] Feng T. H., Jia Q. J., Meng X. (2020). Evaluation of genetic diversity and construction of DNA fingerprinting in *Polygonatum* Mill. based on EST-SSR and SRAP molecular markers. *3 Biotech*.

[18] Dai J., Shi X. D., Gu Y. X. (2017). Development and function analysis of SSR markers in *Magnolia officinalis* transcriptome. *Chinese Herbal Medicine*.

[19] Li C., Zhu Y., Guo X. (2018). Transcriptome analysis reveals ginsenosides biosynthetic genes, microRNAs and simple sequence repeats in *Panax* ginsengC. A. Meyer. *BMC Genomics*.

[20] Liu Y., Fan Z. H., Sun H. B. (2011). Analysis of SSR information in EST resource of *Pharbitis nil*. *Chinese Journal of Pharmacy*.

[21] Liu Y., Zhang P., Song M. (2015). Transcriptome analysis and development of SSR molecular markers in *Glycyrrhiza uralensis Fisch*. *PLoS One*.

[22] Wang D., Cao L. Y., Gao J. P. (2014). Analysis of SSR locus information in *Codonopsis pilosula* transcriptome. *Chinese Herbal Medicine*.

[23] Wang L., Wang Z. K., Chen J. B. (2015). De novo transcriptome assembly and development of novel microsatellite markers for the traditional Chinese medicinal Herb,Veratrilla *bailloniiFranch* (Gentianaceae). *Evolutionary Bioinformatics Online*.

[24] Zhuo L., Xiang C. L., Xiao J., Ye Y. L. (2021). Application progress of SSR markers in plant germplasm resources identification. *Contemporary Horticulture*.

[25] Huang G. W., Guan T. Q., Zhao Y. Y., Chen M. L., Liu H. H. (2018). A rapid and efficient method for extracting DNA from *Camellia oleifera* leaves. *Molecular Plant Breeding*.

[26] Guo Y., Lin H., Liu Z., Zhao Y. H., Guo X. W., Li K. (2014). SSR and SRAP marker-based linkage map of *Vitis vinifera* L. *Blotechnology & Blotechbologlcal equlpment*.

[27] Powell W., Morgante M., Andre C. (1996). The comparison of RFLP, RAPD, AFLP and SSR (microsatellite) markers for germplasm analysis. *Molecular Breeding*.

[28] Song C., Liu Y. F., Song A. P. (2018). The *Chrysanthemum nankingense* genome provides insights into the evolution and diversification of chrysanthemum flowers and medicinal traits. *Molecular Plant*.

[29] Song L. X., Li G. Q., Jin C. Q., Gong S. P. (2019). Whole genome sequencing and development of SSR markers in *Apocynum cannabinum*. *Journal of Plant Genetic Resources*.

[30] Liu W., Zhang Q. Q., Shu F., Cai Y. L., Ma X. L., Bian Y. B. (2019). Genome-wide SNP/Indel analysis and the construction of genetic linkage maps based on Indel markers of *Morchella importuna*. *Mycosystema*.

[31] Kalia R. K., Rai M. K., Kalia S., Singh R., Dhawan A. K. (2011). Microsatellite markers: an overview of the recent progress in plants. *Euphytica*.

[32] Varshney R. K., Graner A., Sorrells M. E. (2005). Genic microsatellite markers in plants: features and applications. *Trends in Biotechnology*.

[33] Xu Y., Cai N. H., Kang X. Y. (2012). Development of EST-SSR markers and their distribution in woody plants. *Chinese Agricultural Science Bulletin*.

[34] Zhan H. X., Wang Y. L., Du C. H. (2020). Development of SSR molecular markers based on the whole genome sequence of *Glycyrrhiza uralensis.* L. *Molecular Plant Breeding*.

[35] Wang S., Ge X. X., Kong W. Y., Chen H. W., Liu K. F., Wang S. L. (2018). Genome survey and characteristic analysis of SSR in Slvia splenden. *Journal of Beijing University of Agriculture*.

[36] Hong W. J., Hao Z. X., Liu K. J. (2019). Development and identification of SSR markers based on *Punica granatum* L. genome sequence. *Journal of Beijing Forestry University*.

[37] Wu M., Du H. Y., Liu P. F., Liu P. F., Teng J. (2015). Characterization of genomic microsatellites and development of SSR markers of *Eucommia ulmoides*. *Forest Research*.

[38] Fang X. M., Huang K. H., Nie J. (2019). Genome-wide mining, characterization, and development of microsatellite markers in Tartary buckwheat (Fagopyrum tataricum Garetn.). *Euphytica*.

[39] Gong J. Y., Liu H., Zhang X. M., Sun W., Li F. (2018). Characteristic analysis of microsatellite sites and primers development in sequences of *Rhododendron* spp. *Molecular Plant Breeding*.

[40] Zhou T. H., Ding J. X., Tian W., Wang J. (2017). Genomic microsatellite characteristic analysis and molecular marker development for *Gastrodiaelata* BI. *Acta Botanica Boreali-Occidentalia Sinica*.

[41] Gur-Arie R., Cohen C. J., Eitan Y., Shelef L., Kashi Y. (2000). Simple sequence repeats in Escherichia coli: abundance, distribution, composition, and polymorphism. *Genome Research*.

[42] Kumpatla S. P., Mukhopadhyay S. (2005). Mining and survey of simple sequence repeats in expressed sequence tags of dicotyledonous species. *Genome*.

[43] Qiu L., Yang C., Tian B., Yang J. B., Liu A. (2010). Exploiting EST databases for the development and characterization of EST-SSR markers in castor bean (*Ricinus communis L.*). *BMC Plant Biology*.

[44] Botsein D., White R. L., Skolnick M., Davis R. W. (1980). Construction of a genetic linkage map in man using restriction fragment length polymorphisms. *American Journal of Human Genetics*.

[45] Wang S. Q., Wang L. R., Liu S. (2018). Construction of DNA fingerprint database based on SSR marker for *Polygonatum* varieties (lines). *Molecular Plant Breeding*.

