# Supplementary material for: SSR Loci Analysis in Transcriptome and Molecular Marker Development in Polygonatum sibiricum
Source: Biomed Res Int. 2022 Mar 8;2022:4237913. doi: 10.1155/2022/4237913 (PMC8923796; doi:10.1155/2022/4237913)
Supplement: Supplementary Materials — Table S1: information of 500 randomly selected primer pairs. [file 4237913.f1.docx]

Table S1 Information of 500 randomly selected primer pairs

| Marker name | Motif | Forward primer sequences (5′→3′) | Reverse primer sequences (5′→3′) | Product Size(bp) |
| --- | --- | --- | --- | --- |
| P1 | (A)18 | GGGAGAAAGGGAAAGGGCTC | GAGGGAGGCCTTCAGGAAAC | 179 |
| P2*▲ | (A)19 | TCGATGTGGCTCATGCTGTT | GGATGAAAGCATTGAGAATTGAGC | 263 |
| P3 | (A)12 | GGGTGTCATGAGGATGTGCT | AGCTGCCTAATTGGAAGATACCA | 237 |
| P4 | (A)12 | TGCTCTCGATCACACAACCC | CGCTCGGTTTTAGCTTCTCC | 248 |
| P5 | (T)18 | TCTTCCTCAAGCTGCCCATG | TTCCCTGTCCACTTTCAGCC | 268 |
| P6*▲ | (C)12 | CTCCACCACCCATTTCAAATCC | GAAATGTCGGGTGGCCAGTA | 230 |
| P7*▲ | (AT)7 | GGACACAAACACAGCGACAC | TGTTTTCAAATCTTTTGGAGTCTGA | 205 |
| P8 | (AG)16 | TGAATATCCTCGCCGTCAGC | GTCATTTTGCGTCGATCCCG | 155 |
| P9 | (CT)6 | GGTGGGCTAATCAGATGCGA | AACCAGTATCGCACCTCACG | 225 |
| P10*▲ | (GT)10 | GGACACAGGTACAACGCAGA | AAAGAATATGATTGTGCACACGA | 258 |
| P11 | (GA)7 | GACCCTCAGAAACACCCGAG | CGGGAGTAAAGGATTAAGGAAGTGA | 260 |
| P12 | (GA)6 | CCGGCAGCGAGATCGAAA | CCAATCGACCTCTCGTTCCC | 209 |
| P13* | (AG)7 | TGCCAGGAAGAAGGAGAAGC | AAGCAAGCCCAAGGAGCTAG | 222 |
| P14* | (TTA)5 | GGTTCGAGATGGACGTCTGG | AACGAAGTGGACCAACTCCC | 248 |
| P15* | (GGA)6 | GTGCGAGACTGATGGAGAGG | CCCCATCGCCACCATCTATC | 228 |
| P16* | (CAT)5 | AGCCTTTCTGTAACAAGCCCT | TGTACTTCACAGGCGTTCCC | 171 |
| P17*▲ | (ATA)6 | GCTGCTTGAGCTCAGATACT | TGCTTAAGGGTGCACTGCTT | 218 |
| P18 | (CTC)5 | GGATCGGGGAAATGCACTCA | CCACTACGCCGAACAAAACG | 248 |
| P19 | (CAG)7 | ACTCCTCCATCCCTCACCAA | GCAGGACGAGGATGATGAGG | 154 |
| P20* | (GCC)5 | TACCTGCAACAAGACGGCAT | TCTCCTCCTCTCCTTCCAGC | 232 |
| P21*▲ | (AAAT)5 | CCGGTTGGACTTGGATTCGA | ACCTCGCTGTTAGCTGTCAC | 216 |
| P22*▲ | (TTGT)7 | GTGCAAAGTGTGATGATGCTCA | GCCCCATGGAAAACAAAGGG | 253 |
| P23 | (AAAT)5 | CTAGTCTAAGGCCGGCAACC | TCCCTCCACACCTTGAAAAGT | 255 |
| P24*▲ | (TTAA)6 | ACCCAACCCCAAACATCGTT | CTGGAGATCAGAGACGACGC | 273 |
| P25 | (AAAT)6 | ACGAAAAGGGGGCAGATGTT | AGCCATTGGAAACGTGTTGC | 116 |
| P26 | (TTAT)5 | CGGCCTGCTAGAGAGAACTC | AGTAGGAATGGGTGTGGGGT | 261 |
| P27* | (AAAAG)5 | ATTCGAGAGGTCTCAAGCGC | TTGCAGCACATCCAGGGATT | 243 |
| P28*▲ | (ATCGA)5 | GCAACTACTGCCGCTAGTGA | CGAAAACCCCTCGGATCTGT | 127 |
| P29 | (CCCGT)6 | CCGAGGGGATGCGAGCTG | AGGTACTCGAGCAGCTCCTG | 222 |
| P30*▲ | (AAAAG)5 | ATTCGAGAGGTCTCAAGCGC | TTGCAGCACATCCAGGGATT | 243 |
| P31*▲ | (ATCGA)5 | GCAACTACTGCCGCTAGTGA | CGAAAACCCCTCGGATCTGT | 127 |
| P32 | (TTTTA)6 | TCATCCTCCCCTTCATCCGT | GTGAACAACCCAAGCAGCAG | 179 |
| P33 | (TAATCC)6 | GCTAGGGTTTTGGTGGCTCT | AGCACACCAATTCACTCTGT | 226 |
| P34 | (GAAAAA)5 | AGCCTCCATCCCCTAAGTGA | GACGGAGGACAGAAGAAGGC | 113 |
| P35*▲ | (GAAGGA)5 | CTTTCAGCCGAAATCGCCAC | CGATCCCTTGTCCACAACCA | 119 |
| P36*▲ | (GTACCC)5 | CCCGAATTTGACCTGACCCA | TGGACCGTACTCGTCCTGAT | 170 |
| P37 | (AGAAAA)6 | CCGGAGAAGCTCAACTGGAA | TCGTCTTCAGTCCGTTGAGC | 121 |
| P38 | (GAGGCG)7 | AGGAGAGAGGGAGGACGAAC | CACTCCCCTTTCCCAATCCC | 207 |
| P39 | (TC)6acgc(CT)6ccccccga(TC)6 | GAGGGCCTAGTCGAATTCGG | GGCGGCGTGAGAGAGATC | 143 |
| P40*▲ | (AG)6aaaccct(AG)7 | ACCTCCCCTTTCTTCTCTCTCT | ATCTCTCCTCCCATTGCCCT | 119 |
| P41 | (TA)8(GA)7 | GTGATTGTTGGGAGCTTGTTCC | TCTCTCCGCGGATTTAGGGA | 258 |
| P42*▲ | (TC)7gctctctctct(CA)7 | TCAAGGAGACGAGGGAGAGG | CTGAAAAGCCAGATGCAGAGC | 213 |
| P43* | (CT)10aaacccctctctcttgtttggcatcttgaggtggg(GA)6 | GGCACCTCTTTTGCTGCAAA | GGCATTTCCCATGAAAGATGCT | 260 |
| P44 | (AG)10aagtgtgagggtgagttgatc(AG)6 | AGTGGCAAGATGAAAGGGGG | GCACCCGCAACCTTACTATA | 113 |
| P45*▲ | (T)10(TGG)5* | GCAACGACAAGTACCAAACCA | CCTGTGCTGCATCTTGTGTG | 266 |
| P46*▲ | (TC)18(TATC)6(TC)7* | GAGCAAGCATGTGCCACCTC | GGAGATAGCGTGAGTGGTCG | 275 |
| P47* | (TG)6(GTG)5* | CACCTTCTAATGTGGACCCCA | CACACCCTCCTACACAACCC | 211 |
| P48 | (TTA)6(TA)6* | GCTTTGCCTATGTTGCACGG | TATGCCACATGGGTCATGGG | 208 |
| P49 | (TA)8(ATA)10* | CTGCCTAGGCTGTTTGGACA | GGCGAAGGGCTCAGGAATTA | 247 |
| P50 | (A)10(AAT)5* | TCACCATCATATTCCTTCTTAGCT | ACAAGCCTATGGTGTCCTGC | 220 |
| P51 | (A)13 | AGGCTTGATCCAATGCCTGT | TGTGTGGGATGGGTTTGCTT | 272 |
| P52 | (A)14 | AATAACTCCGGCGACAGCTC | GCCGAAGAACAGGTTTGGGA | 228 |
| P53 | (T)12 | AAACAGGGGACTTCAGCTGG | AACACAACTGATGCCACTGC | 260 |
| P54 | (A)13 | ATTTTCAAACGCCGCCTGTG | TCTCATTGTGGCCGGTTTGT | 276 |
| P55 | (A)17 | TAAATCCTTGGTCGGGGCAC | TTTAATGGAGAACGGCCCGT | 224 |
| P56 | (A)15 | TTCAAGGGGTTAAGGGCAGC | TCTTGGATTGACTAGGTGGTTGG | 186 |
| P57*▲ | (TC)6 | TCGTGGACGTCGACAACATT | AGCACTTGTCCGTATTGGCA | 247 |
| P58 | (AG)15 | CTGCCATCCAACTCCACTGT | AACTGCTGACATCTGAGGCC | 209 |
| P59* | (GA)7 | CGTTGTCATCGCTCCACAGA | CGTGCTCAACCCTCTCTCTC | 170 |
| P60* | (TG)6 | CTCCGGCCATGTGTGTATGT | GCCTGAAGAACATGACCATGG | 178 |
| P61*▲ | (AC)9 | AACAAAGGTGGTGGCTCTTT | AATCTTCGGCCATGTGTGTA | 149 |
| P62* | (CA)6 | TGAAGAACCGATGCAAGCATG | TTGTGAGGCCTGAGAAGCAG | 161 |
| P63*▲ | (TG)7 | GAGGTGATCTCGGCATCCAG | TACACCCTGCTCTCTCCCAA | 180 |
| P64* | (GCA)6 | CCGCCACCAGAAACCCTAAT | GCTCATGGACTTGGGGCATA | 262 |
| P65*▲ | (CAG)5 | GGAAAAGGACCAGCCCAAGA | CTCTCATAGGAGGCTGGGGT | 208 |
| P66*▲ | (GAG)5 | CCTCTCCTCTCCCACTCCAA | TGAGAATAGCCGCCCGATTC | 270 |
| P67 | (TTA)5 | ACTGCTTGAATTTCCCAAGGT | TCCCAGACAAAAGGCTGACC | 148 |
| P68 | (AGA)6 | TAGACACGTGGCGATTCCTC | GTGTTGTCTTGAGCTGCAGC | 270 |
| P69 | (TTA)7 | ATGCAAGGATCATGTGGCCA | TCCGGTTCCTCTTCTCTCCA | 159 |
| P70 | (GAG)5 | AACAGTGCCGTTTGTGTGTG | TGCAGTTCCGTTTTCCCACT | 220 |
| P71*▲ | (GAAG)5 | CGTTCCCGTTCATCATTGGC | AGACTCCCCAAAGTTTTCCCA | 148 |
| P72*▲ | (AAAT)5 | GGGGAGACAAAGTTGCCCTT | GCAGTTAGAGATCGAATGTGTGC | 274 |
| P73 | (GTTA)5 | GTAACGCCCCAACCAACAAC | TGGATGATGAACTGTCTGACCG | 199 |
| P74 | (GGTG)6 | TCCGTCGATTATGAGCCAGC | CTATCGCCCCCATGTGAAGG | 257 |
| P75 | (GATG)6 | GGCTTATGGAGGATGGACGG | GTCCCACCTCCATCTTCTGC | 192 |
| P76* | (TTTG)6 | CCAGGGTTTTTGTAGCAATCCC | CCGGCGAAACCCTAGAGATC | 203 |
| P77* | (GCAAG)5 | GATAACGAGGAGGGCGAACC | GTTACAGTGAGTGCTCCGGG | 245 |
| P78 | (CGGGC)5 | CACTCTTCCCGGGATGCC | CTTCTCGTCCTCCCTGGGA | 106 |
| P79*▲ | (GATCG)5 | TGGGTTTGGTTGCTGTGAGT | CACCAACCCATGTCCCAAA | 200 |
| P80 | (AATAA)5 | ACACGGGAGCACAACTTTCA | CCAACCGTACACTTCCCACA | 263 |
| P81 | (TTTTC)5 | CACTAGTGGGCCTGGGTTTT | ACTTCACAACTTGCTTTATAGTACAA | 157 |
| P82*▲ | (GCTGA)6 | CCGCGCCTCAGTAAAAAGTG | AGCCTTCAAACGCCTCTTCA | 242 |
| P83 | (CGGCAC)6 | CCCGCCTACGAGGAGATCTA | GAGGAGAAGCTTCCGCAGAG | 260 |
| P84* | (CGATGA)5 | CGGCTGGTTCTTCTTCTGGT | CTTCACCCTCTGCATCTCCC | 208 |
| P85*▲ | (CAGGAG)5 | CAGAGTCCTCCAATCTCGCC | GCTCCTACTCCTGCTCCTCT | 111 |
| P86* | (CGACGC)5 | CCACCCTCCTCAAACGAGTC | CTGTCGCTCTCTGCCAAAGA | 209 |
| P87 | (CTCCAC)5 | TCCAAAGCTCCAACACCTCC | GAGGTGAAGGAGGTGGAGGA | 217 |
| P88 | (CAAACC)5 | CCCTAATGGATGTCCCTCGC | ATGTCGAAGTCGCGAGATCC | 228 |
| P89* | (TC)7gcaatctacgcgattc(CT)6 | TACACGCACCCTAGGTCTGA | TAGTGGCGGAGAGAGGTGAA | 164 |
| P90*▲ | (TC)9t(TC)17 | GCTCCTCTCCCCCAAAACAA | AGGAAATTGCGGAGGTCTGG | 215 |
| P91 | (A)10cccaag(A)10gaaaaag(A)10 | CCGCCCTCCCTTTTCAAGTT | TGCTGCTTTAGGGGCTTGAA | 255 |
| P92 | (TA)6(GA)7 | AGGTTGAACGGTTCGGATCA | AATGGGATGCCACATCACCC | 183 |
| P93 | (CTC)5ac(CCT)9 | CTCTGCTTGCTCCGCTCG | ACACCACCACATCGTAGAGC | 132 |
| P94 | (CT)6gcctctcttgttctctcctgatgccgcctctctttctctctgc(CT)6 | AAATCCGCCCCGCTCTAAAA | CTAGGCAGCGCTAGGGTTTT | 275 |
| P95* | (A)13(AGC)7* | AGAGAGGTTGGATTGCATTGGT | GTGAAGTGGGAGGTGGGAAG | 181 |
| P96 | (TTA)6(TA)7* | TACTGTTGAGCCACTCTCGC | TCTAAGGGGGAAAACGCACC | 109 |
| P97 | (AT)8(T)10* | CCCATGTGACACCCATGTGA | TTGCTAAGGATCAAGGGCGG | 168 |
| P98* | (TTG)5(TGT)11(TTG)5(TG)6*tttgtttgtaattttcgcac(T)10 | AGAGCACTGCTGAGAAAGGT | GCTCATCTGGCCAAAAGGTC | 275 |
| P99 | (T)15(TG)6* | CCCCCTTTGATCACAGAGCA | AACCACACTGAGACGACGAC | 255 |
| P100 | (ATT)6(T)12* | TCTTGAAGCTCTAGGAACAAGACA | AGAGCTCTAGTGTTTGTGTGCT | 191 |
| P101* | (A)12 | ACAACTTGCGATGTGAGGCT | TCTCGGCCTTTTTCGCTCAT | 256 |
| P102* | (T)14 | GAATGGGCTGCTGTCTCAGT | GCATCTCTGCGCAATACCCA | 160 |
| P103* | (T)13 | GCTAGGCACCCCCTTAAGAC | TGCTGGATCCTTGCTCCATC | 252 |
| P104* | (A)15 | GCAATGTAGGACTTTGGCCAC | GAGGCGGGCTACAGATTTCA | 195 |
| P105 | (T)32 | TAAGAGCTTCCCGGCAACAG | ACAATGAATACTATGCTGTTCGC | 105 |
| P106 | (T)14 | GCTGGCATCTCATACACCCC | CGCTAGGGTTCACTCAAACC | 247 |
| P107 | (AT)8 | AATGCATTCCGAATGGCTGC | ACAGTACACTGATTGGAGGATAACT | 181 |
| P108*▲ | (AC)8 | TGCACAAGCTCAACCTTTGA | AGGACGTGTGCATCTGTAGA | 248 |
| P109*▲ | (TC)15 | ACAGGACTCACTTCCATGCC | CTCTGCCTTCAAGCACTCCA | 198 |
| P110* | (AT)6 | CATTGGCTGGTGAGTGCAAC | AGACCCCGAGCTTGTAGGAT | 213 |
| P111*▲ | (TC)15 | ACTCTCTCTCCCCGACTACG | AGCAGTCGAGGAGAGAGAGG | 147 |
| P112* | (AG)10 | GCGTGTGCAATGTATCCACC | CCTCACTTCAATCCCTAAACACC | 233 |
| P113*▲ | (CT)9 | CGTCGCTCCCCTTGATTTCT | TGAGACTTGGTGGTCAGGGA | 169 |
| P114* | (GAA)5 | CGGAGGTTGTGGAACAGAGT | CTTTCACATGTGTCGCGACC | 109 |
| P115* | (AGC)5 | CTACAGGGGTCCCAGAGGTG | CCATAGCAGCCTCCCAAGTC | 119 |
| P116*▲ | (ATG)5 | ACGTGCTGCTCAAATGCAAG | CTCTCTGCCAGCATGCCATA | 258 |
| P117*▲ | (ATG)5 | GCACCTCATTCATCATCGTCG | TACTGCTGGGCTTGGTCTTG | 185 |
| P118*▲ | (GCT)5 | GGACTACAGCCATTGTGCCT | ATCGATGTTCTCCCTTGGCC | 251 |
| P119 | (TTA)6 | GGATACGGAGATAAGCCAAGCA | AGGCTATGTTGCTGAGCCTG | 122 |
| P120*▲ | (GAT)5 | GATTCGAGAGGGCACAACCA | GGGCTGACAGGATTCCAGTG | 227 |
| P121 | (TTCA)6 | TCTCTAACAGCCGATCACCA | CGCGAACAAGCTGCAGATAC | 203 |
| P122*▲ | (ATTT)5 | GGGAGTAGAAGATGCAAGCGT | AGGTATTGCGTCACGGGAAG | 240 |
| P123*▲ | (GGTG)5 | TGTTGTTGTGGAGGGTTTGG | CAGACACTCCACTCGGACAC | 145 |
| P124 | (AGGA)5 | AGTGGCTACAGACGCTGAAG | ACAAAGCGAGGGGGAGAATG | 240 |
| P125*▲ | (GCAC)5 | AGGAAACACCCTCTCGCAC | GACTCCGGAAATCCTGCACA | 258 |
| P126* | (ATTC)5 | ACATAGGCCCCTCTTTCTTCC | CGGAGGAAGGAAACTGACCA | 273 |
| P127*▲ | (AGAGG)5 | AATTGTTCGTCGGCGGAGAT | GTCCTGTAGGAGTCGGAGGA | 194 |
| P128* | (ATAAA)5 | CCCTCCAACATGAATGGGCT | TTCGGGAATACGTACCCTGC | 158 |
| P129*▲ | (GTTTG)5 | CTTCGTCTTGGGCCATGACT | CTCCACATGCCCATGGTTCT | 244 |
| P130*▲ | (AAAAG)5 | TCGGGGTTTAAATCGTTTTATTTGGA | GCCTAGACCCACCCTGTTTC | 273 |
| P131 | (CCGAG)5 | CTTCAGCACCAGGGAGAGC | TGTTGACGATCTTGGGGTCG | 227 |
| P132*▲ | (CCAAC)5 | AGATGGCATTTCCTCACGCA | CCGCATGGGCAATACAACTG | 231 |
| P133 | (AGAAAG)5 | TGTCAACTGAACACAGCAGAGA | AGCATTGATGAGGGGGTTGG | 139 |
| P134 | (TCGGCA)5 | ATTAGGTTACCGGCCAAGGC | TCCAATTAGTGCTGCCGTGT | 224 |
| P135*▲ | (GGCGGT)5 | ATAATGGTGGAGGCAAGCCC | TTCCCTCTTCTTCACGTGGC | 264 |
| P136*▲ | (ATCGCC)5 | TGCTCTAAATTCGAGGCGCA | GGGTTTGGGGGTCTTGTTCA | 218 |
| P137* | (GGAGGT)5 | GGGTGGAGGGAAAACTACCG | CGTCACCCCCTTCCTCTTTC | 134 |
| P138 | (GATATG)5 | TGTCTGCCTCAGCTATGATGC | TTGGGGGAAAACGACTTGGG | 181 |
| P139 | (TCC)6tctcagatttgttccattatat(TA)9tttttacacacacgcacatacgcatatctatatactataattattggtacgga(ATAG)5 | TCCTCTCTGGAAAAGAAAGCTCT | CCACTGTTCCCCTGCATCTT | 278 |
| P140*▲ | (CTG)6atgatgatggttatggttgttgttagaccaggatgtggatgactcgtc(GCT)7 | AACAGAGCCCAGCTGATTCC | GCGGCTCTCCTCCAACATAA | 278 |
| P141 | (AT)11(AC)6 | CGACTGTAGATGGAGGCGAC | GGGTATTGGATTTCCTACCCGT | 244 |
| P142 | (AT)10tctct(TA)6 | ATCCACACCCGATTCGTGTC | ACTTGGGTTAGACATGGGGA | 261 |
| P143* | (GC)6a(CACACC)9 | GACTAATCCACGGGCCTGAC | TGAAATTAACACGTCCACCACA | 274 |
| P144* | (CAT)6caac(ATT)5 | GGGCAAATCTTCATCGCCAC | CCAGTGCCCATGTGGACAAT | 109 |
| P145 | (A)10(AT)11* | AACTGGAGACTGGAGAGGCT | TTGAAGGACATGGGCTGACC | 238 |
| P146 | (C)10(CA)20* | ATTTCCACTCCTCCACCCAC | CCACCTAGGAGCCACATATGG | 141 |
| P147 | (A)15(AAAC)5* | CCTCACAAGTTCTCAGCCGT | ATAGGTACAGTTCGAGCCGC | 230 |
| P148 | (A)14(AAT)5* | GCCCCTCACGTTTCATAGCT | TCGAGGACGCGAATCGATTT | 189 |
| P149*▲ | (A)12(AAT)7* | TGCAAACAAAGCAGCAACATCT | TGGCCCTCTCTTACTAGCGT | 147 |
| P150 | (TAT)11(TA)9* | TCTTTTGCGACTTCGGTGTT | TCTCAAAACGTGGCCAAAGC | 237 |
| P151* | (A)18 | GCCCAAGAATACAAGCAACCA | GGATCTGTTAGCTTCCGCCA | 139 |
| P152 | (A)14 | GGTGAACGGGGCTGTTATCA | ATCTTCGGCTGTTCTGAGGC | 178 |
| P153*▲ | (A)21 | TTCATGGGCAGAGACTGTCG | TCGAACACCGCTTTGAGACA | 277 |
| P154* | (A)12 | GTTTGCCTGATTCTGATTGAGGA | GGGGGACAGACAGACACAAG | 210 |
| P155 | (A)14 | CCACAAGGGAATCGGTCAGT | GGCAAGGATTCCTACTGCC | 268 |
| P156 | (T)13 | CCTATCAGGCAGGTCCTCGA | GAGCCTTGGCTCATCCGATT | 280 |
| P157*▲ | (G)12 | CAATGGGTCCTGTATGGCACT | AGTGGCAACGAGGAATGGTT | 256 |
| P158*▲ | (T)13 | GCCATTGTGCAGATTCTTTGC | GAGCCTTGGCTCATCCGATT | 177 |
| P159* | (A)15 | AATCCCCATGCTGAGGATGC | GGTTGGAGATCCTTAGATGCCC | 231 |
| P160 | (T)14 | GCAACCGCATTGATGGGATC | CCATTTTCGGTCTCCCACC | 170 |
| P161*▲ | (T)16 | TGCCATGATGCTCAACCATCT | CCATCAACCAGGGACCTCAC | 197 |
| P162 | (C)14 | GGGCACAGAGGAGGAAAGAG | CCACCACGGTGTTAGGGTTT | 279 |
| P163*▲ | (AG)6 | TCCCTGACCACCAAGTCTCA | CCCTTCTCCACCTCAACCAC | 256 |
| P164*▲ | (GT)8 | TGTTGCTGTTCCCGATCTCC | GCTTTTCAGTAACCTCTTCCCA | 214 |
| P165 | (CT)11 | ACCGCAGCTGTTTCTCTCTC | CATGGGAGCTTCTTTGGGGT | 261 |
| P166* | (AT)6 | TCATATCGCCGCAGAAGGTC | CTACCGATTCCGCCAACAGT | 204 |
| P167 | (TC)14 | TTAGTACCTGCCGCCTCTCT | GGATAGAGGGTTTCGCCTCG | 165 |
| P168 | (AG)7 | ACCGCTGTCAATCATCCTGG | TTAGCCCAACCGGTAACACC | 269 |
| P169* | (GA)11 | ACCCCATACCCACACCCATA | CCCTACACCTCCCCTCTCAA | 184 |
| P170* | (CT)19 | TCTCCACTTGCTTGCCATCA | AGCCAACTCTGCCATTGGAA | 239 |
| P171*▲ | (CT)11 | AGAAAAGTGAGGCGAGGAGG | GGTGGCTTTAACAGGGACGA | 109 |
| P172*▲ | (CT)9 | TCCCAAGATCCCTTCCCCTT | ACACTCTCACCGGCCTATCT | 267 |
| P173*▲ | (GA)6 | ACCACCTGCTGATGATGAATGT | TCAAGTCTCAAGCTCCCTCA | 143 |
| P174 | (TC)10 | TTTGAGTTGGGCTGGAGGAC | GCCGAAATGGGGGAAGAGAA | 244 |
| P175*▲ | (AG)7 | TAACCCATGGCTGTCCTGGA | CAAGAGTCCTCAGGAGTTGGT | 270 |
| P176 | (TC)7 | CCTACCTCCCAACCTGCATC | CGACGAATTCGCCGTTGATG | 253 |
| P177* | (GGA)5 | AGCTCTGCTCATGGAAAGGA | GCTGGTGGTGTTGGACTTGA | 188 |
| P178*▲ | (GGA)5 | CCGATGACAGGATCGACGTT | TCCTCACCCACAACACCAAC | 273 |
| P179 | (TCC)10 | AACGCCGCTAAAAGACTGGA | TCTTCTAAAGAGGCGGACGC | 140 |
| P180* | (TTA)5 | AGGCCACCACATTTGATCTGT | GCCGCACAAGTATAGTTGGC | 279 |
| P181 | (TAA)9 | TGGCAAGATAGAATCACCCCG | ACCGTGACATATACTCGGGT | 189 |
| P182*▲ | (CTT)5 | CCCCTCTTCTTCTCTGCTCC | GGTGCTGGACCTGGTGATTA | 277 |
| P183 | (GGA)8 | GACTTGTTGCGGTCGGATTC | AACCCTCAGACCTACCCCTC | 157 |
| P184 | (TGC)5 | CAAAACCCTCCCGCCAAATC | CACAGACACGCTCAGCAGTA | 236 |
| P185 | (CAT)5 | TCTGTCTCCCTGTTCTCCCT | TCGTCAAATCCTTCGTGGGG | 123 |
| P186* | (TCC)5 | ACCTTCTACGACGGCTACCT | GAAGGGGGAGCAGAATCCTG | 206 |
| P187*▲ | (AAT)5 | AAACCACGCATTGCCATCAG | TGACAGCGTCCACTGGATTC | 219 |
| P188 | (ATT)9 | CGCTGCAGTCGATACCATCT | AAAACAGGCGATGCAGAGGA | 260 |
| P189* | (CTC)6 | CGATATGGAGCTGAGGCAGG | GTCCGGGCAATCTCCTCTTC | 155 |
| P190*▲ | (TAT)7 | AAGGGGTGAAGGAGGGATGA | TGGTCGAACAATAACAGGGGG | 262 |
| P191*▲ | (ATA)12 | GACTCCCAATTGCCCTTCCA | TGACTCACGCCGCACTTTAT | 191 |
| P192* | (TTA)6 | GATCGTGTGGAGGTTGAGGG | TGGTTTTAGTCCCACATCGGT | 215 |
| P193 | (TAT)10 | ACATCAAAAAGCAAAAACAAACTGC | TGCATAGGGTTCATGGCGAG | 186 |
| P194 | (CGC)5 | CGTTTCTACGGGAGAACGGG | GGGCGCGGTTTGAGTGTG | 236 |
| P195*▲ | (AAG)5 | CCATGCATGCTCCTCTCGAT | GGTGAAGGTGGAGTTGAGGG | 182 |
| P196 | (TCA)8 | TGCCGAGATACTTCAGGGGA | AAAGGCACGACACTTAGGCA | 139 |
| P197 | (GAA)5 | GGATCAAGACGGCCAAGACA | TCGCAGATCCCAATTTGGCT | 268 |
| P198* | (TCT)5 | GCCATCTTCTCTCTCCTCGC | TACACCACTCCAAACGACGG | 203 |
| P199 | (ATT)6 | GCCTTGAGTAGACACCCACA | AATCATGCCTGCCACTGTGA | 274 |
| P200*▲ | (GGA)5 | CGCCGAGAACATAAGCAAGC | CCGAATCCTAGACGCAGACC | 270 |
| P201 | (C)12 | TGTACTAGCGGTTGCCTGTG | GGGAAACAAATTTTGGGCGGA | 152 |
| P202 | (C)12 | AACAGCTGCTACTGCCTCTG | TGCCATCGAAACATTTTTGCT | 150 |
| P203* | (G)12 | GTGGCATGGAGGAGGAGAAG | CGCACCTTCTCCTACACCAG | 181 |
| P204*▲ | (C)12 | TTCAAAGCCTTGGGTGACGT | GACGAAGGCATGAGGGAGAG | 244 |
| P205 | (G)13 | TAGCATACGAAATGGCGGCA | AGAGTGTGCATGTGAGGGTG | 246 |
| P206 | (G)14 | GCGACTTTGGTGAGGAAGGA | AGCTCTCCAAGATTGCCACC | 177 |
| P207 | (G)13 | GGTTGGAGTTGTGTGTGTGC | TGCCTGTGCATCTCACTTCA | 224 |
| P208 | (C)12 | ATTCCTCCGTGGCAGTAACC | TTTACAAAGCTACCCCCGGC | 250 |
| P209*▲ | (C)14 | GGGAGGGAATGTGGGTGAAG | ACATGCACCCATCCTGTTCA | 271 |
| P210*▲ | (A)14 | TGATTCCTCTTTGTTGCCCGA | CGATCAATGTGCTTGGCGAG | 226 |
| P211 | (AT)11 | TAGGTGAGCTGTCAACGCAG | CCGATTCAGCCGATTCCGAT | 204 |
| P212 | (CT)19 | TCTTGGCAATCCTCTCTCGC | AAAGCCCAAAGTGTTGCACC | 210 |
| P213 | (TA)8 | AGTGGAAGACTGATTGCCCG | GATGAAAAGGACAGCCACGG | 202 |
| P214*▲ | (AG)7 | GGAGGTTTTTCCCCGTGACA | CTGCGGAAGAATCAAGCGTG | 165 |
| P215*▲ | (CT)20 | GGTTGTAGTTTGTTATCATGCCGA | ATGTTCGGAACAGCACGAGA | 144 |
| P216 | (AT)10 | TAGGCCAGGCAAGAAAGTCG | ACTGCTTCTGGGGGTTGTTT | 231 |
| P217 | (AG)9 | CGACCAAAGGCGGTGTTTTT | GGTGCCTTGGAAGACAGTGA | 199 |
| P218 | (GA)8 | GGCGAGATCGAGAGCACAAA | GCAAGGAAGGGGAGAACCTC | 106 |
| P219* | (CT)6 | TCGCTACCTCTCTCCTTTCA | GAGGGCTTTGGTTAGGGCTT | 140 |
| P220*▲ | (GCT)5 | CCAAGTCTCTGCATCCACGA | TTCTCTGGTCGCCGTCAAAA | 248 |
| P221*▲ | (GCC)5 | CAGCAAGCAACAAGAACCCC | GCGAATCGGCCGAAAAGTTT | 141 |
| P222 | (AAG)6 | ACCCCCTTTCCCAATTGTGA | CCAACCTGAGCCCATCATCA | 105 |
| P223 | (CGG)8 | AGCTTCTGCAAGAGCCTCAG | TCATGGAATCAGCCGCCTAC | 255 |
| P224* | (TTA)5 | AGGTTTTGACCCCTTCACTCA | TCCCAGCGAAGACCAAGAAT | 280 |
| P225*▲ | (TTA)5 | CTGCGGAGGATAAGTAGGCG | CGCGATTTCTCCAGATTCGC | 271 |
| P226* | (AAT)7 | AATGCTCCAGTGGCACCAAT | CGAGCCCGAGCCTAAGATG | 244 |
| P227 | (TGC)6 | GGGATGTTCACTGACAGCGA | TGCCGCTTGGACTTCTTCTC | 236 |
| P228*▲ | (TAA)6 | ACCACTCTCTGACTCCCTCTC | CTCTCGCAGAACGCCTCTAG | 259 |
| P229 | (GCC)5 | TCCTCGTCGGGGGATAAAGA | GGAGGAGGAGGAAGAGGAGG | 279 |
| P230* | (GAT)5 | ACCCCTAGACACCTGAGCAA | GCCGCTCGGGTGTTTTCA | 274 |
| P231*▲ | (AAG)5 | TTCATGGCGTCTTCAGACCC | GAAACAGGTTGCTGCCTTGG | 266 |
| P232 | (TTC)5 | CAGGGTGCATGATCCAGAGG | CCTCCTCCGACTCCTCTCTC | 263 |
| P233 | (ACA)5 | GACAATTCAACAAGGCCTGCA | TCTGATGTTGTGGCTTTTATGTCA | 135 |
| P234* | (GCA)5 | GGGGTTTGATGCTCCAGGAA | GCCTAATCGTCTCGGTCCAG | 142 |
| P235 | (AAG)6 | AGTCATTAGAGGAAAAAGAAGAGGGA | CCTCTTGAGACCCGAACACC | 253 |
| P236*▲ | (TTA)7 | GCCTTGCAAATGCCGAGAAA | CCATTCATGCGAGGGAGGAA | 165 |
| P237* | (GAA)6 | GGCATCTCTCCACGATCACC | GACGAAGGCGAGGATGAGAG | 266 |
| P238 | (GGC)6 | GCAACGCAACGAACCTTTCT | GGAAGAGGAGTGACTGTGGC | 123 |
| P239*▲ | (CAG)5 | TGTTTGCAGTAGCCCACCAA | AAGATGGGCGGCTGCATAAT | 249 |
| P240* | (ATA)5 | GCATTTGAGCTCCAACGGAC | AGTCATCCATTCACCGAGGC | 223 |
| P241*▲ | (AAG)5 | CCATGTGGCACGTAGGACTT | GGGTGGTTACAGGAGATGCA | 260 |
| P242*▲ | (CCG)7 | TTGAGACTTCGGACTCGCTG | TACATGTACATGCCCTGCGG | 277 |
| P243 | (GCC)5 | TCCTCCCTCTTCTGCCAGG | CTACATCGACCTGCAGGGAG | 279 |
| P244* | (TCC)5 | AACTCCACTCCCTCCTCTCC | TCGCTCTGGAGTAGGAGCTT | 169 |
| P245 | (AAG)5 | GACAAGGGCCACTCTTCCTC | ATCGCTGTAGCTCCTCCTCA | 250 |
| P246*▲ | (CTG)5 | CAGGATCAGCTTCGCATTGC | ACCATTGCCAGGCTACCTTC | 141 |
| P247*▲ | (GGT)5 | ATCGAACGGAGATCAGCGTC | TCACCTTCGCCTCCCTTTTC | 199 |
| P248*▲ | (AGA)6 | GAGAGGAGTTGGGGTTTGGG | CATCATGAGCGAGCCTAGCA | 214 |
| P249 | (ACC)5 | TCCAATGTCATCGGTTGCCA | GGATCGACACGGTTGACTGT | 262 |
| P250* | (CCT)5 | CTCTCCTCCCACTCCCTCTC | GACGACTTCGCCGATCTAGG | 212 |
| P251 | (AAG)7 | GACCGGTGATTGGAGTGATGA | TGGAGCTCAGCCTCATCAGA | 280 |
| P252* | (ATT)6 | GAGCACACCAGAGGAACACA | TGCTCAAATTTGCCACATGC | 247 |
| P253* | (TTG)5 | ATTGGGCGTCCTTGATGGTT | ACGAACGAGAGCTGCTGAAA | 209 |
| P254* | (AAT)5 | GGCTGTGTTTGGTTTGAAAGGA | TCCCAAAACCAAGAGAAAGTGAGA | 255 |
| P255* | (ACC)5 | TACAACCAGGCACAAGGTCC | AGTCCCGAAAGAGAATGCCG | 251 |
| P256*▲ | (GCA)7 | AGGGCCGTGCTCATAGATTG | CGTCCTTTCCAATCTCAATTCGT | 276 |
| P257* | (CTC)5 | CGTCCATGGCCTCTCTTCTC | GGAGGGTCGTTAGTTTCCGG | 252 |
| P258 | (ATCC)5 | TCCAGGCCAAGAATGACAGAC | TGCCTCACAACAAGAAGCCA | 129 |
| P259 | (TTCT)8 | GGCAGTTGCTAACAGTCATGC | ACAGAGGCTTCCATGGCATC | 176 |
| P260*▲ | (CATA)6 | GTGCCGTCCTTGAAGTACCA | TCATGGTTACACCCCTGCAC | 159 |
| P261* | (ATTA)5 | TGATAGGAGGAACTTTGCCACA | AGACATTTCCACCATCTGCCA | 126 |
| P262*▲ | (CTTT)5 | CAGATCGCGAAGAATCCGGA | GAAGAGACATGGGAGCCACC | 257 |
| P263* | (TGCA)5 | GTTCAGGTTCTGGATGCCGA | CCGATGAGAAGCCCCATACC | 270 |
| P264*▲ | (AAAG)7 | GACTTCGAGCTACGGGGAAG | GACCCCACCCTTCCAAACAA | 255 |
| P265 | (TAAA)5 | AGCCGCCTAGAAGACACTTG | AGCTTATGAGGATGATGACTAGGT | 239 |
| P266*▲ | (TAGA)7 | AGCAATTAACTTACACTTACTTGCCT | CTGTGATGATCAGCAACGCG | 279 |
| P267* | (AAAT)6 | CATGCTGGTAGTGCCTGGAA | CATAAACAAGCCGAGCTCGC | 248 |
| P268*▲ | (TTTA)6 | TGGCGATCCTCCGATACAGA | AGGGTTCAGGCTTCACACAC | 258 |
| P269*▲ | (AAAT)5 | AGCTCGAATCACGTTCACATCT | TGGTGTTTGGGTAAGACTGCA | 193 |
| P270 | (TATT)5 | AAAGGAGAGGGTGATGCCAC | CCAGAACCAGAACCAGAACCA | 134 |
| P271 | (TTAT)6 | GCCTTATCCCCTCCCCCTTA | TGTGGCTGAGCAAGATGGAG | 266 |
| P272*▲ | (ATAA)5 | CTTGGGAGCATTCTAGGGTCA | TCAATTTGTGGCTCGTTCACG | 280 |
| P273 | (ATTA)5 | AGAGTGCAGTTTGGATACGCA | GTGCTAATCGCGCCAATCTA | 154 |
| P274*▲ | (TTCT)5 | ATTTCAGAAGCCCCCACCTT | GCAAAGGCTCCAGTGTCAGA | 246 |
| P275 | (AAAT)5 | TGGAAGGCCTCAGCTTCAAG | ACCCGAAAAACATTGGCACG | 266 |
| P276 | (GACA)5 | GAACGTGCTCTGCTGGAAGT | TGTGTCCATCTGTCCGTGTG | 253 |
| P277 | (AAAT)6 | GAGCAAAACTCAGTGATCTAAGGA | AGCCTTGTTGCTGCTACCAA | 109 |
| P278*▲ | (TGTA)6 | GATTCGCTAGCGCAAAACGT | ACCAGAACCAAGTAAGCAGCA | 254 |
| P279 | (CTCG)5 | CTCATTCGTCCTCTCATCTCCC | AAAAACCAACGCGAGAACGG | 178 |
| P280 | (TTAA)5 | TGTTTTAGCAACACTTGGGACA | GACCAGCCCCTTTGCATTTC | 228 |
| P281 | (TTTA)5 | ATCCTCGCATGACCCTGATG | ACACATCACTTCTTGTCGAGACA | 262 |
| P282*▲ | (GAGC)5 | GCCCAGCAGAAAGAGAGGAG | TTGAAGAGCAGCAGGAGTGG | 147 |
| P283 | (TTGA)5 | TGAGTCCATGATGGGGGCTA | CTGGCCTTGTTCTGGAGGAG | 141 |
| P284 | (TTTA)5 | CCTTTGCATGTATGGCACGG | GTGTTTAGGCCGAGAGCACA | 227 |
| P285 | (AATT)5 | GCACATAACCATATGACCAGCC | CAACTGCATGTGGCCTTGAC | 181 |
| P286*▲ | (AAAT)5 | TGTTGTAACGGATCCGCGAA | GTGCGCGCGATTGGAATTTA | 195 |
| P287*▲ | (AAAG)5 | ACCCTAGTCTCGATCGCACA | TCCTCCCATGGACAAGTTGC | 274 |
| P288*▲ | (AAAT)5 | GTAGATCAGTGCTTGCCGCT | GGCTGAAACGCCCAAACAAT | 274 |
| P289 | (TAGC)5 | TCTCTTGTTGCTCGGTGGTC | AGAAACACAGACAAGGCCCA | 102 |
| P290*▲ | (ATTC)5 | TTCCTCGTCCTCATCCTCGT | CCGCCAACACGAACAAACAT | 178 |
| P291 | (TCTT)5 | AGCCTCAATGAAGGCGTTGT | TCCATTTCAATCAGTTCCAGAACA | 189 |
| P292 | (AATT)5 | CTAGTCAACCCCATAGAAGGCG | GCAACTGCAGGTGACCTTGA | 100 |
| P293 | (TTTA)5 | TCCTATGCATAAGCGGGCTG | ACAAGCAACAACTTTTCTCGCT | 250 |
| P294 | (TCCG)5 | CTCCGCAGATTTCCCCCTG | GGAGGAACCAACCCTTAGGC | 160 |
| P295*▲ | (AAAG)5 | ACACTTGGGCCTTCTTTGGT | GGGAGAGAAGATTTGAGGCCT | 147 |
| P296* | (AGGG)5 | TGACACAACAACCACAACGA | TTCCCCTGCTTGAAGACACG | 269 |
| P297 | (TCTT)5 | CTTTGGGAGAGCAGGAGGTG | CAACTCAGGAGACCAACCCC | 104 |
| P298*▲ | (TTAT)5 | AAGTTACGATGATAGTTAACAAGTGAT | CTGGTGCCAAGGTCGAGAAT | 231 |
| P299 | (AAAT)7 | CAGCACAAGTGTCACCCTCA | ATCGATGACTGGGTGCTTGG | 143 |
| P300*▲ | (TATC)7 | CCGGACAGTTCTGTGTGTCA | AGCTCTGATACCATGTAGAGAGA | 191 |
| P301*▲ | (AAAT)5 | AACCTCTTGATTCAGCCTTGT | TTTGCCATCGAAGAGGCCTT | 173 |
| P302 | (CTTT)5 | GCAACTACCCAGCCACCAAT | TCCTGGCACTTTCATCAGTGA | 276 |
| P303 | (GCCT)8 | ATTCCTGCAGCCCTTCAGAC | CCTCTGCACCGTCCTCAAAT | 269 |
| P304 | (AAAT)5 | TTGCAAGCCCCTGTTTGTTG | ACATTCCTATGTGCGACACTACA | 268 |
| P305 | (AAAT)5 | TGGGCTGTTTTCACGTAGCT | GCTCGACCTTGTCAATGTTGT | 278 |
| P306 | (ATTT)5 | ACAGATGGTTAATCATAGAGAAGATGC | ACTGAATTCATGCTCGCCGA | 109 |
| P307* | (AAAG)5 | GCCCTCCATGTGAGAGAGTG | ACATTGCGCCACAAATTGCT | 190 |
| P308 | (TTCA)5 | AATCAGACTCTGCCAGGTCG | TGCTGCTTCTTGGAGTTGGA | 125 |
| P309*▲ | (AAAT)5 | TCGCCTTTGCAAGCCTTCTT | CCCCTACGATAATGCCCACC | 245 |
| P310 | (TATT)5 | CCCTGTCATACTCACGTCCG | AGCAATATGTTACTTTATGCCACCA | 121 |
| P311 | (GGAT)5 | CGGCGACTCTTTAGGTGGAG | CATGTGTCCTCCCCCAACC | 125 |
| P312 | (ATTT)5 | TCAGTCGATAATTGCGCCCA | GATCGTTGCATCGACTTGCC | 267 |
| P313 | (TTTA)5 | GAGCACCAGTACTTGTGGCT | AGGCTGCCTTTTTCCTCCAA | 244 |
| P314* | (GATT)5 | GACAGGGTAGCGCTGACTAC | TCTTGGGTTCGCTGATGACC | 161 |
| P315*▲ | (ATGT)5 | CTACAGGCGGGTGCTGAATT | ACAAAATAGGTGGCTGGCCT | 264 |
| P316*▲ | (AAGA)6 | GAAACGGGTGTTAATGGCTGT | GATGACAAGTGCGTGCATCC | 187 |
| P317 | (ACAT)6 | AGCTAACGTGGCTAGAGTGC | ACTTTAACGCTCCGCTGGAA | 117 |
| P318 | (TTAT)5 | AGAAAGTGAAAGCCCCATCTCT | AGCTGCCTCAAGAATGGACG | 233 |
| P319*▲ | (TTCA)5 | CCCCCTTCGCTTTCTCTGTT | GCCCAGCCCTCAAAGTTTTG | 164 |
| P320 | (TTTA)5 | ACTGGAATGAACAGTGGGCT | GTGTGTTTGACGGGTGTTCG | 214 |
| P321 | (TTTC)5 | GGTGCTTCAAAGTGCCAAGG | AGCGCCAGAGAAAGGTGATC | 136 |
| P322 | (AAAT)5 | CTCCCAAAATTCAACCCAACCA | GAAGGTGAAGTGCAATGGCC | 144 |
| P323*▲ | (AAAT)5 | ATGTTGCCGTACACGTGTGA | ACCGAGCGTCAAATATCAAAGC | 221 |
| P324*▲ | (TCAC)5 | CCGGGGTACACTGCATCTAC | GTGATTGCCACAAAGCAGCA | 227 |
| P325* | (TTAT)5 | CTCGCCGACAACTTAGTGGA | CATCCAGCTCCTAACAGGCC | 267 |
| P326*▲ | (GAAGG)5 | AGCTTCTTTGATTCATAGAACAGCT | GTCCTAGCAGCCACATGGAG | 264 |
| P327 | (AAAAT)5 | GCCTTGTTGCCTCCGTTATC | GTAATTGGAGCCGCTACCGA | 215 |
| P328 | (AAAAT)6 | TGGGCTTGCTATCAGACACG | TCATGTAGCACGGGTTGGAA | 164 |
| P329 | (ACTGT)5 | GGGCAACAAAGGCTTCTGTG | AAGCAATGGTGAGGCTCTCC | 214 |
| P330 | (AAACA)5 | TCACACACGTACAGGAGGGA | GCTGAATTCCAGGTGTTGCC | 154 |
| P331 | (CCCGT)5 | CCAAGTCTGGTCTAGCAGCC | AGCAGCACCTCATCGATGC | 266 |
| P332* | (AAAAT)5 | CGGGACCTCTTGAGTCACAC | AGAGGAAGGATCATGGGAAGA | 161 |
| P333 | (GAGAG)5 | TCTCTCTAAAGCAACAACAAAGCA | GAGGAGAACGAGGGCTATGC | 227 |
| P334*▲ | (TTTTC)5 | ATTCGTGTCTGAAGTGGCGT | CACGTACGGGTTCTATGCGA | 267 |
| P335 | (TCTTT)6 | AGGCAGAAACTTGATATCGCCT | ACACTTTCTCTTAAAAAGGAAACAAGA | 272 |
| P336*▲ | (GAAAA)6 | CGTCGATACCAACTCCGAGG | ACCCCTGGTTGAGTAGTATAACT | 143 |
| P337 | (GAAAA)5 | GCCACTCCTGTCAAAATGCA | CCAGCAGTCCATCTTCAGGT | 204 |
| P338 | (AAAAT)6 | TTGATGCGATTTGATTTGGTGA | GGTGTAATAGAAGCCCGGCA | 114 |
| P339 | (CCTCT)5 | AAGAAGAGCAGACGCAGGTC | GAGGAGAGATTGGGATCGCG | 174 |
| P340 | (TCAAC)6 | CCTCGTTGTCCATGTCCACA | CACTTTCGGGACTCAGCACT | 264 |
| P341*▲ | (CTTTG)5 | TGTGATTCAGCAGCTGGTGT | TCTCGCAGCCCAGATAAAGC | 240 |
| P342* | (TAAAG)6 | AAGCTCGTTAATCAGGCCCC | CGCGAGGGAAGTCTGTTTCT | 190 |
| P343 | (AAATA)6 | GGCTGATTGTTGAGATCCGGA | CCATCTCCTTTCTTCTTCCATTTT | 280 |
| P344*▲ | (ATTTT)5 | CTAGGGGCAGGGATTTCAGC | TGCCCCCAAAACAGAACACT | 184 |
| P345*▲ | (AAAAG)5 | TGGTTTGTCACCCTGCAAGA | AAGCAAACCGTACTGTTGCG | 254 |
| P346 | (CGCCT)5 | CTTCACCTCAGCGCCGCT | GGTTGCAGTAGGGGCAGTAG | 280 |
| P347 | (GGAGA)6 | TGTTCCATCCCTTGTTGAGG | AACTGTTGATTCGTGCAGCG | 238 |
| P348 | (GAACC)5 | ATTTGTGGAGAAGGCGGAGG | ACGAAACCGAACCGAACAGA | 231 |
| P349 | (CGGTT)5 | TGGTGTTGGAAAGAATGCGC | TTGGCAAAACCGAACCGAAC | 273 |
| P350*▲ | (TATTT)5 | ACAGTTTGCGATATTAGAGGGGA | ACAATGCAGAATGGAATCAAAGT | 172 |
| P351*▲ | (TATTT)5 | GTAGGGCACAGGGAGGAAAG | GGGTTTAGTCCCACATCGG | 234 |
| P352*▲ | (TGTAT)6 | GCTTCATTCGCCAGCTCAAC | GATGAAGTCCTCGTTGCCGA | 239 |
| P353*▲ | (CACTT)6 | ATGAATGGTGTGGGTGGGTG | TGTCAGAGACGGCAAAAGCT | 247 |
| P354 | (AGAAG)5 | GAGCCTAGGCCAAGTTTCGA | TGCGGGGTGACTGTACTAGT | 235 |
| P355*▲ | (AGAAG)5 | CCTAGTCCTAGGGTTGCCGT | TTTGGTGAGTGTCTCGGTCG | 280 |
| P356 | (AAAAG)5 | AAATCCCACGCCACGAAGAT | TGGTTGTGTGGTGAGTGTGA | 159 |
| P357*▲ | (TTTCT)5 | GTGCTGCTCACTCCCTTCTT | CGACCAAGGTCACTTTCCCA | 247 |
| P358 | (TTTGT)6 | ACGGAATGGGAGGAGATGGA | CCACTCTCACAACGCTCCTC | 258 |
| P359 | (GGGCC)5 | CTCTCGGAGCCTAGCATGG | GAACGGTGTCTCCCCGAG | 248 |
| P360*▲ | (CTTTT)5 | GACGGACATGACGAGGGATC | CGAACACAACAAAGGAGGCA | 144 |
| P361*▲ | (ATTGC)5 | AGCATGCAGAGAGAGGAGGA | TGCATTGGACCTGCAGCTAA | 217 |
| P362 | (CCGCT)5 | GGTTTCCGTTAGCGCCGG | CGACTGCGGCCTACAAGG | 277 |
| P363 | (TTTTC)5 | CCCAGGGACATGGTTATCGG | GCTTTTATCCGCTGCACCAC | 232 |
| P364*▲ | (GATTG)6 | GATGCCTGAACACCTCCACA | GCCAACCAGTCACATTTCTGT | 240 |
| P365*▲ | (AAAAG)5 | ACCCCTGATGAACCATGCAG | CACACTCTGTGGCTTTGCAT | 190 |
| P366* | (CTTTT)5 | AGGAGGTGACAGGTAAGGGA | TGACGGCTATTGTGATGGCA | 267 |
| P367 | (CCGAG)5 | GGGAGGAAGCTGGAAAGGAG | CATCCGACCTCATCCCATCC | 244 |
| P368*▲ | (TTTAT)5 | CAGAGTGAAGCTAAGGTGCCA | AACCTTACCCCCACTCCCTT | 172 |
| P369 | (CGATC)5 | GATACCCATTCGCAAACGCC | TCAGAGGGCAGCGAGAAATG | 280 |
| P370*▲ | (TTTTG)5 | TCCTAGGAGGGTGGGTTACG | TGTGCATTTGGTTGGGGGAT | 174 |
| P371*▲ | (TCCGA)5 | GCAGAACCACCAAGACTCCA | CCTCCCCAAGCATCATCCTC | 278 |
| P372 | (CAAAT)5 | AGCAAACTGAAGCACTGAGC | GGCGATTTGGGTTTTGGTCC | 122 |
| P373 | (CCAAC)5 | CGAACCCCTCCCAACCTAAC | AGTAGCTGGTGCGCCTTTTA | 115 |
| P374*▲ | (TTAAT)5 | AATGACGACGGCCTCTTCAG | CTACCCGATCCGATTCAGGC | 265 |
| P375* | (AAATA)5 | TCATCAAAGGACTCGCTGCA | CCCGCGATCGCTTCAATTTT | 268 |
| P376 | (GTTTT)5 | GCTTGGGGAAATGGAAGGGA | TGTTGTTGTTCCTTAAACCCAAA | 227 |
| P377 | (ATTTT)5 | AAGCTGCACTCTTGTGGTCA | ACTGCACTAATAACTTATCAGCGT | 260 |
| P378 | (AAAAT)5 | GCTGGGTGCGTTTGTATAGC | TCCCCATGGCATTTCAAGCT | 150 |
| P379 | (AAAAT)5 | GCAAGATTGGTGGATGCAGC | TGGAGCTCTGGTGAGGATCT | 276 |
| P380 | (GAAAA)5 | CTCGTTTTTCCACACACGCA | CCACTTTTCTCCTCGGACCC | 269 |
| P381 | (ACTCA)5 | CCAGGCCTATCAAGCAACCA | TGCTTTTTCTGTTTCTTTTATTGAGGG | 239 |
| P382 | (AAGAA)5 | TCCTCCACTCCAGTACTCCG | GGCCATCATCTGCACCTTCT | 217 |
| P383 | (CCGGG)5 | CTGCATAGGGGAGGGGAGG | AACTTCCGGTCGCTCACTTC | 137 |
| P384 | (TTTTG)5 | CTTTCAGCACAGGGAGAGCT | ACCCCCTCTCCCTATTCCAA | 254 |
| P385* | (TCGCC)5 | AATCATCGGAGGACGGCATC | ACCGAAGCCAAGCAGAGATC | 181 |
| P386 | (GGGAG)5 | GCTATGGGAGCGTTCAACGA | ATACGCTCATAGACCGCTCA | 245 |
| P387 | (GAGCC)5 | CAAAACCGTGGGGAAGGGG | AGATTTATCACCGCGCCTCC | 120 |
| P388* | (TCAGA)5 | TCTAGGCTGCCTCCTCCATT | CCTACTCCTCCCCTGAGCTT | 254 |
| P389*▲ | (AAAAG)5 | ACCCATACTCGTGTCTGCTC | AGCATGCAGGAGAGTGAAGA | 217 |
| P390 | (AATGC)5 | GCTGCTTAATATTAGGGCATGACA | AGATAGATTTGGACGTCCATGG | 167 |
| P391 | (GCCGT)5 | CCCAGAAGAAGTCGTGTGCT | CAGGAAACGTCACCTCGGG | 232 |
| P392* | (TTATT)5 | GAGCAAGGACGTTCAGCAGA | TGTCTAATACAAAGAAACCCACCA | 276 |
| P393*▲ | (TTCTG)5 | GAAAAGACTCGAGCCCTCCC | TCCTGTTGAACCCTGCATGA | 148 |
| P394*▲ | (GATCT)5 | TCGATGCAGATGCGGAATCA | GAGGAGGGAGCTTTGATCCG | 274 |
| P395 | (ATTTT)5 | TAATGCTGGTGTGCTGGAGG | GCCTTCTCTCAACACACAGC | 198 |
| P396 | (ACGGG)5 | TTTAGTGGAGGCAGAGGGGA | CCCGATCCCATCCCGAAATT | 230 |
| P397 | (GTTTG)5 | TGATTGTCCGAGTGAGTTGC | CTCCACATGCCCATGGTTCT | 276 |
| P398* | (TATTT)5 | CCTTGATTCTCACGGCGGAT | ATCCCGATCGATCAAACCGG | 252 |
| P399 | (GGAAGA)5 | CGCTAGTGGAAATGAAGCATGG | GTTTACCCCTTCCTCTGCCC | 111 |
| P400 | (AGGGAG)5 | ATCCTGGTTGCGGAAGAAGG | CAACATCACCTCCCCCTTCC | 201 |
| P401 | (CAAATC)5 | CGCCGTCACTCTCTTTGTCT | GTGGGCTGTTTGAGAGAGCT | 226 |
| P402* | (GATTGG)5 | CTTTCATGCATCTGTCGCCG | TTCCTCTCCGGTCCAATCGA | 232 |
| P403 | (AACCCT)5 | TCTTCAACCCCCAATTCCCG | GAGACCTCAGATTCGGAGCG | 224 |
| P404*▲ | (ACAAAA)5 | ACGAGACCATCTTCCCTAGCA | TACAGAGGGGGCCAATAGCT | 237 |
| P405*▲ | (TGTGTA)5 | TCGACAAGTATCCGAGTTTCCA | GCTCCACAGTCAACCCCATT | 235 |
| P406 | (CAGCAC)5 | ATCTGGGATCAGGTGGGGAA | GTTTATCAAACAACTCCGGAACG | 210 |
| P407* | (GGCGGA)5 | CCAACCCTAGACAGACAAGACA | CGTTGATGAGTCGTCCACCA | 124 |
| P408* | (CGTGGC)5 | AGGTCCATTCTGCTCTTGCC | ACTACCACGGCCACCTCTAT | 176 |
| P409* | (GGGAGA)5 | CGAGGGTCTTGGGAGAGAGA | AACTCGACCCGAACCGAATC | 243 |
| P410 | (AAAAAT)5 | CGCGAAATGAGCCAGCAAAT | GGAAAGCTGTGGTCAAAGTTCC | 116 |
| P411*▲ | (ACCCTA)5 | ATGTCCTTCGACTCCTCGGA | AAGTAGGGCTTCCGGTTTGG | 230 |
| P412 | (GGAAGA)5 | CGCTAGTGGAAATGAAGCATGG | GTTTACCCCTTCCTCTGCCC | 111 |
| P413 | (AGGGAG)5 | ATCCTGGTTGCGGAAGAAGG | CAACATCACCTCCCCCTTCC | 201 |
| P414 | (GGAAGA)5 | CGCTAGTGGAAATGAAGCATGG | GTTTACCCCTTCCTCTGCCC | 111 |
| P415 | (CGGGAT)5 | GGTCTTGCTCTTGCCCTTCT | CATAGCCCAGAGAGCGGC | 280 |
| P416*▲ | (TCGATT)5 | CATCGCATCATCAGCAGCTG | GTACCAAGCGAACAACACCG | 237 |
| P417 | (TATGAA)6 | ACCAGTGGCAGTACTATCCCT | TAGCTGCGGTTATAGCCAGC | 212 |
| P418 | (GAGGCG)7 | AGTGTGAGAATGGTGGTGGG | AGGAGTTACAACCCCATCCCT | 263 |
| P419* | (ATGCAA)5 | TACGCACCCCATCCTACGTA | TTGCATGATGTTGCGTGGTG | 176 |
| P420*▲ | (AACAAG)5 | CAAACTTGCCCAAACCCTCG | TTTGCAGGCGGTAGAACCAA | 242 |
| P421*▲ | (CTCTCC)5 | CAGTGCGTGCATTGTGTGAA | TTGTTGTCGACGCGAAGAGA | 255 |
| P422 | (CTGCTA)5 | AAGAGCATGGGTGTGATGGG | CATGCACGCACCAAGAGAAC | 193 |
| P423 | (GGTGGG)6 | GGTGGAGGTGGAGGTGGTAT | CGGTAGTTTTCCCTCCACCC | 208 |
| P424 | (CCACAC)5 | CAGCACCCAAAACACCACAC | TGACCCAGAATGCCGCAAAT | 187 |
| P425*▲ | (GCACCG)5 | GTGAAGGTGAGCGTCTCCTC | AAATCCGCCGTCAGCTTGAA | 177 |
| P426 | (CGGGGC)5 | GAGGGGACGGGGACAAGAA | CTACGGGGTCACGGTGAG | 187 |
| P427*▲ | (GGAGGC)5 | GGGAGGTAGAGGAGCTTCGA | ACAGGGCATAGGCATAACGG | 158 |
| P428* | (ATTTTT)5 | TTGATTTGAATGCCGTGCCG | ACGCAAGATAAGGAAGAGCACA | 237 |
| P429 | (AGAAAG)5 | TGTCAACTGAACACAGCAGAGA | AGCATTGATGAGGGGGTTGG | 139 |
| P430 | (CGGTGG)5 | GGCTCTTCAGCTAGGGGTTC | GCCAAATTCCAACCCGAACC | 188 |
| P431* | (GGGGCG)5 | CCGGGATGATCTGATCGGTG | GCCCTCCACCTTCCTTTCTC | 254 |
| P432 | (TTGGCT)5 | TGTGCAGCATGGTGTTTTCG | TTCGAAGAGCACCAGCACTC | 271 |
| P433 | (TCAACT)5 | TTCCGTTCGAAGCTCGTCTT | AGGAGGGAGATGAGCTCTGG | 184 |
| P434*▲ | (TTTATT)5 | AAGTCAGTTTAGGCGGTGGC | CCCCTTTTGCTAGTGCGAGA | 227 |
| P435 | (GGGGGT)5 | CGCCATCTGTTCCCACTACA | AAACACCCAACTCCAACCCC | 258 |
| P436 | (GTTCGG)5 | CCAGCGACTTCTCCGTCTTT | CGAGCCAATCCTCATCTGCT | 122 |
| P437 | (GGGCCG)5 | CTCTGCTTCTTGCTGTCCCA | GTCCTTGTCGCTCTGCAGG | 275 |
| P438* | (TGCACC)5 | TTCACTTCCACGAGTTGGCA | AGGATAAGCGGGAGTGGGAT | 213 |
| P439 | (GGGATT)5 | AGCGACAGATGCTCAACACA | GCCAATCCTCACTCTTCCCC | 220 |
| P440*▲ | (ATCCCC)5 | GAGATCCTTCCTCCGCTTCG | TCCGTCTCCGTCTCCATCAT | 210 |
| P441* | (TCCCAC)5 | TGGCGTGGAGGAAAGTTCAG | CCACTCTCGTTCTGCCCTTT | 231 |
| P442*▲ | (TGGGGA)6 | TGCCATTAGTGAACGCGTCA | CAGCCTTGCAGCAAACCAAA | 195 |
| P443 | (GGGATT)5 | GAGGCGGTGGATTAGAGAGC | TCCATCCCCCTCCTTTTAGC | 104 |
| P444*▲ | (TGGGCT)5 | GACGGCATCGGCAACTTTTT | TTTTTGCAATGCCAGCCCAA | 231 |
| P445 | (ATCCGA)5 | CCTTTAGACATCCGGCCCAA | ATATCCGCATCCGAATCCGC | 232 |
| P446 | (GGGTGT)5 | GTTTGGCTTCGGTTCAACCC | CTGCTTCAGGTCACGAGTGT | 138 |
| P447 | (AGAAAA)6 | CCGGAGAAGCTCAACTGGAA | TCGTCTTCAGTCCGTTGAGC | 121 |
| P448*▲ | (TTAGGG)5 | CTCCTCCTCCTCTCCCTTCC | AAACCTGCTTCCTCAGTCCG | 211 |
| P449 | (GGAAGA)5 | CGCTAGTGGAAATGAAGCATGG | GTTTACCCCTTCCTCTGCCC | 111 |
| P450* | (TTAGGG)5 | CTCGCCACGAAGCTGTAGAT | TCACCAAATCAAGAAGCCAGC | 133 |
| P451 | (GCGCGG)5 | AGTCAAATCAAGCCACAACGG | CCTAACCCTAAGCACCACCG | 251 |
| P452*▲ | (CGGATA)5 | GTTCACGAAGTAGGGGTGCA | GCATCCAATCCGCTAATATCCG | 173 |
| P453 | (TATCCG)5 | AGTATTCGCATCCAATCCGCT | GCCTTTACCATATCCGTATCCA | 145 |
| P454 | (TTAGGG)5 | AATCAGTGAGGAGCCAGCTG | TCCACACAACCACAAGCCTT | 105 |
| P455 | (TTTTTA)7 | TAGCTGGTTCTTTGGGTCGC | TGCGAGAGGTGGTGAGAGTA | 217 |
| P456 | (TATATG)8 | TGCCACAACAAGGTATGGAGA | TTCTTGTCCTTAGCCTCGGC | 206 |
| P457 | (TACTAT)6 | CGACAACAACACCACCAACA | GGCAGACGGGTGATAATGCT | 212 |
| P458 | (CTCTCC)6 | GTTACCTTCGCCCTCTTCCC | TCGGTTTGGTGCTCATCGAA | 132 |
| P459 | (GCGCGG)5 | CGGAAGCAGCAGAACAGAGA | CCTAACCCTAAGCACCACCG | 267 |
| P460 | (TATTGG)6 | TCAGGGGTCGATGTCAGGAT | GCACGCATATTCGCTTGGAA | 277 |
| P461 | (GGGGGT)5 | GGCGGAAGAGGAGGAAGAAG | AAACACCCAACTCCAACCCC | 201 |
| P462 | (GAGAGG)5 | TGTGGGAAGGAGGAGGAGAG | GGGAAAACCTCACCATCCTCT | 154 |
| P463*▲ | (CACCTG)5 | CAGTTGCAGCTCTCCACAGA | CTCCCTCCCTCGAATCGTTG | 221 |
| P464*▲ | (TCATCC)5 | CCAAACGTCTTTCGGCAACA | GTGGCTCAGATGCAAGACCA | 237 |
| P465 | (GT)7atg(T)10 | TGCCTTTCTGGTGGTAAGCT | CGGCAAGCAAAATCTTTTCACG | 248 |
| P466 | (GC)6(AC)9 | CGGGTGCCCAAAACAGGTAT | TGGAGATGCTGCACTTCGAA | 201 |
| P467 | (A)15(TG)7 | GCCAAGACAGAGGTCAGGTG | CTACACCCCGCAATTCTGGT | 247 |
| P468* | (T)11(GA)7 | TGCAGCTAGTGTACTTGCCT | ACAGGGTGACTAAAGTTGCC | 198 |
| P469*▲ | (T)10gtgatactggatggagcgaatccccacccgccaag(A)10 | GGACAGGGCTCAATCTAGGC | GTGGGGTTGTTCACCTGCTA | 278 |
| P470 | (A)10tcacactaa(T)10 | TGAGTTGGAGATTGAGTTGGGA | TCGGTTGCAGAGAATGGCAT | 214 |
| P471*▲ | (A)10tcacactaa(T)10 | GGTTGTCGAGGGGTCGAAAT | TCGGTTGCAGAGAATGGCAT | 253 |
| P472 | (AT)6agcttggggaggggag(T)10 | TGGTCCACCACATCCAACAG | CACGCGGTCCATCTTATCGA | 147 |
| P473 | (A)16tttaggg(T)10 | ACTCGGTCCAGTCCATCGTA | GGATTGGCCCGTGACACATA | 227 |
| P474 | (T)10ctttagatg(T)10 | TGAGCTTGGTTCGATCTGGG | TGCAGGGGTAGAGAGGTCTC | 235 |
| P475* | (T)11ctcaactttgtcaaactgggtcgggctttcaatggcccatacgtgggcca(T)10 | TGGCAGAAGCAGTGTCAGAG | GCCTCAAGGTGTATGTGCCT | 279 |
| P476*▲ | (AG)6aagggtgcaaagcatgtcacctcacctatttaattttagccctcacctgttctt(CA)6gaggagg(GA)13gggatcaagaaaaggaaaacaatttgtatagatagat(AG)6tgagagagagaa(AG)9 | ACCTCCCTCAGTGGACAGTC | ACGCTAGGGATGTGCATAGC | 270 |
| P477*▲ | (T)11ctatttttgtaaatttctttttgtggaagcatgaatgtgaggagtatttcagaagcctcaacaatgcttctctgattttgatgtagatgagagagaaagt(AG)8 | ACAACCACCCTCGTTTCCAA | TGAAGACCCCAGGAGCAGTA | 253 |
| P478 | (GA)11gctgagggattaggg(T)11 | TAACTTACCTGTCGAGGGCG | ATTCCCAATTGACCCGACCC | 251 |
| P479 | (T)12cctat(A)11 | GCAAAACTTTATTAGGTCAATTGCGT | TGGCGTGAAAGAAACAAGCG | 182 |
| P480* | (GA)7a(AG)7 | AGAGGGATGAGAGATGGGCT | TCCTCAAATATCTTTCTTCTCTAGCA | 184 |
| P481 | (CT)7(AT)7 | CTTCACCGCCACATGTGTTG | CAGTGTGCCCTCCTTGTTGA | 276 |
| P482 | (AT)6gag(AT)8 | AGGCCGAGGAAAAACGTCAT | ATCTCTGTGCGCTTCTGCTT | 249 |
| P483*▲ | (AAT)5aagcaaaag(A)10 | CCACCACCCACCAGAAGAAT | CCCCTAGTTCCCTAGCTCGT | 150 |
| P484 | (CA)7tg(TA)7 | GGCAGAATCCGTTCCTGGAA | AATCCCCATCTTCCAGTGCG | 268 |
| P485 | (AC)6aa(AC)9 | CTTGACCTTTCGGCAACTGC | ACACAACTCCAGGAACTCCAG | 251 |
| P486 | (C)10gcgcctcat(C)10 | AGAAGTCCAAAGCCGACAGC | TGCAGATTTGGGGAGCTGG | 176 |
| P487 | (A)14tcaaacaaaaaacccc(A)10 | ACGATTCCAATTCTCTCCCCC | GGAGAGGGTTTGGGGATTGG | 116 |
| P488 | (AC)8(TC)6 | CTCTGCGGGAGTTGTAGTGG | TGTCCTCCTACAAGTGTTCAAA | 224 |
| P489*▲ | (T)12gga(TG)7 | TCCTGGTGGAGTAGGCTTCA | TCAGAATCCCCAGGCTCAGT | 132 |
| P490 | (TC)6gatctctctcgaaacctctcagaacccta(TC)7 | CTGAAAGGCAAGGGCAGAGA | AGATCGGGCTGAGAGAGAGG | 260 |
| P491 | (A)10tg(A)10 | CACAGACCGCAGTAGCTTGA | GGGAGACTGAAGCCATTGGT | 279 |
| P492*▲ | (CT)7gtctctg(TC)8 | ATTCCCAGGGCACATGTGAA | GCATGCCCAACAAATCCCAG | 260 |
| P493 | (GA)7gt(AG)7 | GTGCCTGGTTAGCTCGAGAG | TTTATTCGTGACGCGGGGAA | 156 |
| P494 | (A)10cgag(A)11 | TCAGGTTTGGTGTCCGTGAG | TGGACTATGCCATGGCCAAG | 200 |
| P495 | (A)12c(AT)13 | CTAAGGATCAAGGCTGGGGC | GGGATTCCTCATATGCCGCA | 235 |
| P496 | (TA)12(GA)7 | GTGATCGGCACCGTGTGT | GAGGGGTGGGCTCACATATG | 146 |
| P497 | (GAG)5gacgaggaa(GAG)5 | GAGCAGAGCTCGGTGGAG | CAGCGATGAGAGCCTCCAG | 229 |
| P498* | (GA)8gcta(AC)8 | GGGGAGAGAGAGCAGTGAGA | TATTCGAAGGCTGGGTGCAG | 190 |
| P499*▲ | (CTC)6(TTC)5 | GAGCTCCGTGGTTGTTCTCA | ACGGTGTCGGAGAATCGATG | 167 |
| P500 | (CA)9 | CTCTTGCTCTCTCCCTCCCT | CATACATGCATGGGGAGGGG | 140 |

“*”：SSR primer information that can be amplified

“▲”：SSR primer information with polymorphism
